# Statistical Method Based on Bayes-Type Empirical Score Test for Assessing Genetic Association with Multilocus Genotype Data

**DOI:** 10.1155/2020/4708152

**Published:** 2020-05-06

**Authors:** Yi Tian, Li Ma, Xiaohong Cai, Jiayan Zhu

**Affiliations:** ^1^School of Mathematics and Statistics, Central China Normal University, Wuhan 430079, China; ^2^School of Information Engineering, Hubei University of Chinese Medicine, Wuhan 430065, China

## Abstract

Simultaneous testing of multiple genetic variants for association is widely recognized as a valuable complementary approach to single-marker tests. As such, principal component regression (PCR) has been found to have competitive power. We focus on exploring a robust test for an unknown genetic mode of all SNPs, an unknown Hardy-Weinberg equilibrium (HWE) in a population, and a large number of all SNPs. First, we propose a new global test by means of the use of codominant codes for all markers and PCR. The new global test is built on an empirical Bayes-type score statistic for testing marginal associations with each single marker. The new global test gains power by robustly exploiting the Hardy-Weinberg equilibrium in the control population and effectively using linkage disequilibrium among test markers. The new global test reduces to PCR when the genotype for each marker is coded as the number of minor alleles. This connection lends insight into the power of the new global test relative to PCR and some other popular multimarker test methods. Second, we propose a robust test method based on the new global test and the ordinary PCR test built on a prospective score statistic for testing marginal associations with each single marker when the genotype for each marker is coded as the number of minor alleles by taking the minimum *p* value of these two tests. Finally, through extensive simulation studies and analysis of the association between pancreatic cancer and some genes of interest, we show that the proposed robust test method has desirable power and can often identify association signals that may be missed by existing methods.

## 1. Introduction

Association analyses that test multiple genetic markers as a set rather than individually have been appreciated for their potential power. These statistical methods largely fall into three classes: those for summarizing *p* values from the tests of each single marker [1–5], those that synthesize single-marker test statistics, such as Hotelling *T*^2^ (standard Chi-squared) statistic [6–8] and the burden test [9, 10], and those based on a direct test of joint associations of multiple markers, such as variance component tests (VC) [11–13], the sequence kernel association test (SKAT) [14–18], and principal component regression (PCR) methods [19–21]. The relative performance of these methods has been comprehensively compared in previous work [22]. When the number of single-nucleotide polymorphisms (SNPs) is small, these methods have similar power; however, when the number of SNPs is large, the effects of SNPs are not constant and may have different directions, the linkage disequilibrium (LD) among multiple markers is somewhat strong, and the SNPs adopt additive genetic code. Three methods, namely, VC, SKAT, and PCR, have been found to have competitive power in this case [22, 23]. A major reason is that all 3 methods can decrease the degrees of freedom of the test to some extent [12]. In this work, we focus on exploring a robust test for unknown genetic modes of SNPs of interest, unknown Hardy-Weinberg equilibrium (HWE) in a population, and a large number of SNPs of interest.

We first propose a novel multi-SNP test under the case-control study design, which we term the principal Chi-squared test. The principal Chi-squared test applies a two-degree-of-freedom score statistic based on the empirical Bayes method for each SNP and derives a global test based on the eigenvalue decomposition of the asymptotic variance-covariance matrix of each SNP test. The global test achieves improved power by robustly exploiting the HWE in the control population and effectively exploiting the LD among all SNPs. We denote the global test by PChiB (see Methods). In addition to competitive power, PChiB is conveniently implemented and is easily comprehensible by the nonstatistics community because of the well-known eigenvalue decomposition method. The global test is closely related to standard PCR in that it reduces to the score test of PCR when each SNP is coded as the number of minor alleles. This relation not only lends insight into its power relative to PCR but also into the connection between PCR and variance-component-based tests. We show that both classes of these methods are weighted combinations of uncorrelated Chi-squared random variables, each of which is a weighted combination of a single SNP test with weights equal to the loadings of the eigenvectors of their joint asymptotic variance-covariance matrix. This observation, while supporting documented conclusions that none of the two classes of methods is uniformly more powerful than the other [22], reveals theoretically that the LD structure among SNPs plays a critical role in the powers of these methods. When a real disease causal SNP adopts recessive and dominate codes, test PChiB can gain desirable power. When a real disease causal SNP adopts an additive code, test PChiB may have somewhat lower power. Thus, we propose a robust test by taking the minimum *p* value of the new global test PChiB and the ordinary prospective score test of PCR in which each SNP is coded as the number of minor alleles, regardless of the actual genetic code of each SNP. We denote the robust test by Min2.

Suppose that *q* diallelic SNPs in a genomic region of interest are genotyped for *n*_1_ case samples and *n*_0_ control samples. Let *Y*_*i*_ denote the binary case-control status (*Y*_*i*_ = 1: case; *Y*_*i*_ = 0: control) for sample *i* (*i* = 1, 2, ⋯, *n*), where *n* = *n*_1_ + *n*_0_, the first *n*_1_ samples are cases, and the remaining *n*_0_ samples are controls. Denote *G*_*ik*_ as the count of the minor alleles of SNP *k* from sample *i* for *i* = 1, 2, ⋯, *n*, and *k* = 1, 2, ⋯*q*. A new global test is designed to test the null hypothesis that the genomic region spanned by *q* SNPs is not associated with the phenotype status of interest against the general alternative that one or more SNPs, which may or may not be genotyped, are associated with the phenotype status of interest. We fit an ordinary logistic regression model for the binary case-control status and all SNPs.

Incorporating HWE constraints into the control population based on the retrospective likelihood for testing a diallelic marker may lead to increased power under dominant and recessive genetic models compared to standard prospective likelihood-based tests [24]. To address the issue that deviation from HWE may lead to an inflated type I error rate in this test, an empirical Bayes score test, which is a data-adaptive linear combination of the prospective likelihood score test and retrospective likelihood score test under the HWE constraint, was proposed [25]. This test can maintain nominal type I error rates under deviations from HWE that are observed in real settings and largely maintains the power gain under the recessive genetic model. Here, our new global statistic uses this test principal as the building block. We expect that our method achieves considerably improved power when aggregating the small power gains at each SNP.

The rest of this paper is organized as follows. In Results, we demonstrate, through simulation studies and analysis of pancreatic cancer data [26, 27], that the proposed robust test can often have desirable power compared to some popular tests across a broad range of scenarios. In Discussion, we further discuss the merits and disadvantages of our proposed test method and note some directions for future research. In Methods, we present the new global test in detail and discuss its connections to PCR and other existing methods. We also briefly introduce the robust test by taking the minimum *p* value of the new global test and the score test of PCR, where each single SNP is coded as the number of minor alleles, regardless of the actual genetic code of each SNP.

## 2. Results

### 2.1. A Robust Statistical Method Based on Two Types of Principal Chi-Squared Tests

For real genotype data, we can first calculate the prospective score test, denoted by ${\tilde{U}}_{P}=\left({\tilde{U}}_{P,1},\cdots ,{\tilde{U}}_{P,q}\right)$, in which all SNPs are supposed to adopt additive codes. We denote a consistently estimated covariance ${\tilde{V}}_{P}$ for ${\tilde{U}}_{P}$ and calculate the ordinary principal components regression (PCR) score statistic, which is denoted by *PChiP* (selecting the top PCs explaining 85% of genetic variability) based on the estimated covariance ${\tilde{V}}_{P}$, as in Gauderman et al. [19]. Second, we can obtain the *p* value of PChiP, which is denoted by *PV*_*A*,*P*_ because PChiP follows a Chi-squared distribution asymptotically under the null hypothesis. Third, we calculate the empirical Bayes score denoted by *U*_*B*_ = (*U*_*B*,1_, ⋯, *U*_*B*,*q*_) and its consistently estimated covariance, which is denoted by *V*_*B*_, based on codominant codes (see Methods). Similarly, we calculate the new aforementioned principal Chi-squared statistic PChiB based on the estimated covariance *V*_*B*_. Note the dimension of *U*_*B*_ is 2*q*, and we can estimate the *p* value of PChiB, which is denoted by *PV*_*C*,*B*_, because PChiB also follows a Chi-squared distribution asymptotically under the null hypothesis. Finally, we take the minimum of the two *p* values of PChiP and PChiB as a robust test, as follows:

(1) $$\begin{array}{c} \mathrm{Min}2=\min\left(PV_{A,P},PV_{C,B}\right). \end{array}$$

We estimate the *p* value of Min2 via statistical permutation. We conduct extensive simulations to investigate the power performance of Min2.

To view the performance of Min2 comprehensively, we can compare it to 4 other tests, namely, PChiB, PChiP, SSUP (see Methods) and GOLD, where GOLD is constructed as follows. Suppose the first SNP is the real causal SNP satisfying the logistic regression model logitPr(*Y* = 1) = *β*_0_ + *β*_1_∗*G*_1_, where *β*_0_ and *β*_1_ represent log odds ratios. Other SNPs are correlated with the first SNP with genotypes *G*_2_, ⋯, *G*_*q*_. The Gold method (denoted by GOLD) is an ordinary score test based on the above real statistical model. Clearly, in real data analysis scenarios, we do not know the causal SNP. GOLD only has a value in simulation studies and is not practical in real data analysis. We consider 3 scenarios for analysing genotype data. First, we apply PChiB, Min2, PChiP, SSUP, and GOLD to analyse genotype data, including all SNPs. Second, we apply PChiB, Min2, PChiP, SSUP, and GOLD to analyse genotype data, excluding the first SNP, which is the causal SNP. Third, we apply PChiB, Min2, PChiP, SSUP and GOLD to analyse genotype data, including only labelled SNPs. To comprehensively assess the performance of these 5 methods, we designate every SNP among all *q* SNPs as the causal SNP in turn in the simulation procedure.

### 2.2. Simulation Procedure

We conduct extensive simulation studies to assess the relative power of Min2 by comparing its performance with that of 4 other test statistics, namely, PChiB, PChiP, SSUP, and GOLD. We consider real LD structures defined by haplotypes inferred from the International Hapmap Project CEU samples. We set the haplotype information for gene NAT2 studied by Kwee et al. [28] as the basis of our simulations. To generate multilocus genotype data based on real haplotypes, we estimated haplotypes and their frequencies in a genomic region via HaploView software [29]. The LD structures plot based on the complete set of SNPs for gene NAT2 is displayed in Supplementary Figure 1 (See Supplementary File). For gene NAT2, we select SNPs with MAFs > 0.05 and genotype rates ≥ 75%, for a total of 18 SNPs. Haplotypes based on the complete set of SNPs and their frequencies are provided in Table 1. Five SNPs, rs13277605, rs1799930, rs1208, rs1961456, and rs2410556, are tag SNPs.

To obtain the *n*_0_ control samples, we generated multilocus genotype data, as follows. Let {*f*_*H*_} denote the set of estimated haplotype frequencies with ∑_*H*_*f*_*H*_ = 1. Then, a pair of haplotypes for each control sample was generated under HWE, where the frequency of haplotype pairs (*H*, *H*′) takes the form *ϕ*_*HH*′_ = *f*_*H*_^2^ as *H* = *H*′ and *ϕ*_*HH*′_ = *f*_*H*_*f*_*H*′_ as *H* ≠ *H*′. The haplotype phase information was then deleted, and only locus-specific genotype data were retained. To generate multilocus genotype data for each case sample (total number *n*_1_), we generated the pair of haplotypes (*H*, *H*′) using the following probabilities:

(2) $$\begin{array}{c} \phi_{HH\prime }^{1}=\frac{R_{\text{Aa}}^{I\left(HH\prime \text{includes}"\text{Aa}"\right)}R_{\text{aa}}^{I\left(HH\prime i\text{ncludes}"\text{aa}"\right)}\phi_{HH\prime }}{\sum_{H,H\prime }R_{\text{Aa}}^{I\left(HH\prime \text{includes}"\text{Aa}"\right)}R_{\text{aa}}^{I\left(HH\prime i\text{ncludes}"\text{aa}"\right)}\phi_{HH\prime }},. \end{array}$$

where *R*_Aa_ and *R*_aa_ are the odds ratios for genotypes “Aa” and “aa”, ‘A' is the major allele for the disease causal SNP, ‘a' is the minor allele of the disease-causal SNP, and indicator functions *I*(*HH*′includes^"^*Aa*^"^) and *I*(*HH*′includes^"^*aa*^"^) refer to whether haplotype pair (*H*, *H*′) has allele combinations (A,a) and (a, a), respectively, at the causal SNP.

To evaluate the impact of deviation from HWE on the power of PChiB, we additionally generated multilocus genotype data from real haplotypes based on gene NAT2, as described above, but with the frequency of haplotype pairs (*H*, *H*′) equal to *ϕ*_*HH*′_ = (1 − *F*_*st*_)*f*_*H*_*f*_*H*′_ + *δ*_*HH*′_*F*_*st*_*f*_*H*_. Here, *δ*_*HH*′_ is an indicator function, with *δ*_*HH*′_ = 1 if *H* = *H*′ and *δ*_*HH*′_ = 0 if *H* ≠ *H*′, and *F*_*st*_ is the fixation parameter, which represents mild deviation from HWE, as observed in real gene association analysis studies.

We set *n*_1_ = 1000 and *n*_0_ = 1000 and consider two scenarios with HWE indicator *F*_*st*_ = 0 and 0.5log(2), as in Luo et al. [25]. Furthermore, we designate every SNP as the causal SNP in turn. When the causal SNP adopts an additive code, we obtain the genotype and case-control status based on the logistic model with causal SNP odds ratio 1 for estimating the empirical type I error rates and with causal SNP odds ratio 1.2 for estimating the empirical power. When the causal marker adopts a dominant code, we obtain the genotype and case-control status based on the logistic model with causal SNP odds ratio 1.3 for estimating the empirical power. When the causal marker adopts a recessive code, we obtain the genotype and case-control status based on the logistic model with causal SNP odds ratio 1.5 for estimating the empirical power. With the genotype and case-control status information, we calculate the *p* value of Min2 via 200 permutations. The empirical type I error rates and powers of the 4 tests were considered under a significance level of 0.05 by means of 500 repetitions, as Kwee et al. [28] examined the type I error and power of the semiparametric and single-tag SNP approaches assuming a nominal significance level of 0.05.

### 2.3. Numerical Results

To comprehensively assess the performance of Min2, we construct test statistics under 3 scenarios, namely, using all SNPs, using all SNPs except the causal SNP, and using only tag SNPs.

**Figure 1:**
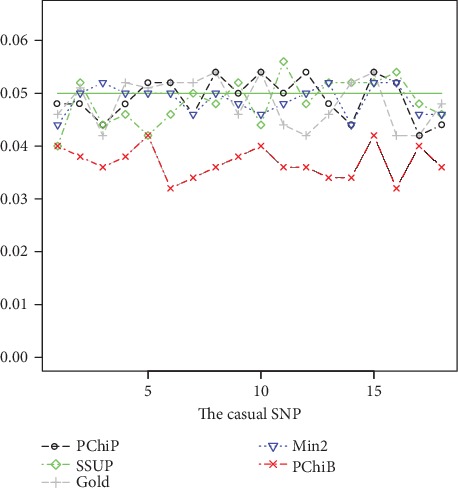
Empirical null hypothesis rejection rates (based on all 18 SNPs) of GOLD, PChiP, SSUP, PChiB, and Min2. Each SNP is treated as the causal locus in turn, which has an additive effect, with simulated odds ratio 1.0 and *F*_*st*_ = 0 based on 1000 controls, 1000 cases and 500 iterations.

**Figure 2:**
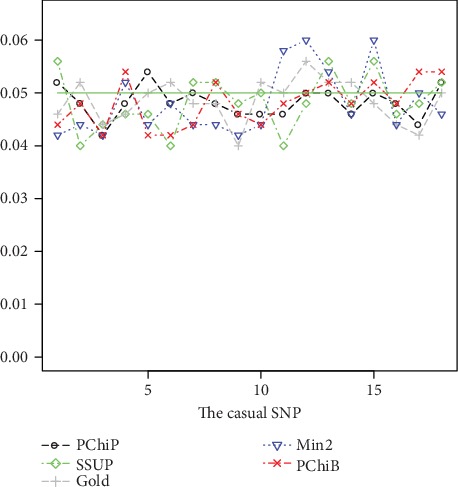
Empirical null hypothesis rejection rates (based on all 18 SNPs) of GOLD, PChiP, SSUP, PChiB, and Min2. Each SNP is treated as the causal locus in turn, which has an additive effect, with simulated odds ratio 1.0 and *F*_*st*_ = 0.5log(2.0) based on 1000 controls, 1000 cases, and 500 iterations.

Because the empirical type I error rates are nearly the same when the real causal SNP adopts an additive code, dominant code, and recessive code, we present the empirical type I error rates for only the case where the real causal SNP adopts an additive code. The results based on all 18 SNPs with *F*_*st*_ = 0 are displayed in Figure 1, and the results based on all 18 SNPs with *F*_*st*_ = 0.05*l*og(2) are displayed in Figure 2. Other results based on all 17 SNPs (excluding the causal SNP) and 5 tag SNPs are displayed in Supplementary Figure 2a, Figure 2b, Figure 3a, and Figure 3b (See Supplementary File). From Figures 1 and 2, we can see that Min2 can control the type I error rate well when the HWE indicator coefficient *F*_*st*_ equals 0 or 0.5log(2.0), but PChiB has a conservative empirical type I error rate when *F*_*st*_ equals 0. We further investigate this phenomenon: when the real genetic model adopts additive code, PChiB adopts a codominant code with *F*_*st*_ equal to 0, so the correlations between every two SNPs are decreased and test PChiB may absorb a large number of degrees of freedom. For example, when considering the scenario with all 18 SNPs and designating the 1st SNP as the causal SNP, PChiP absorbs 2 degrees of freedom and PChiB absorbs 5 degrees of freedom, according to the simulation data. When the real genetic model adopts recessive and dominant codes, all 5 tests control the type I error rate well, regardless of whether *F*_*st*_ is 0 or 0.5log(2.0).

For the empirical power comparison, when the real causal SNP adopts a recessive code, we display the results based on all 18 SNPs in Tables 2 and 3 for *F*_*st*_ = 0 and *F*_*st*_ = 0.5log(2). Other results based on 17 SNPs (excluding the causal SNP) and 5 tag SNPs are displayed in Supplementary Figure 4a, 4a, Figure 5a, and Figure 5b (See Supplementary File). From Table 2, Supplementary Figure 4a and Supplementary Figure 4b for *F*_*st*_ = 0, we can see that the GOLD test always performs best because it is an oracle test, and Min2 performs nearly as good as PChiB in all 3 scenarios. Additionally, Min2 always performs better than PChiP and SSUP, regardless of which of the 18 SNPs is the causal SNP. For example, in Table 2, the empirical powers of PChiP, SSUP, GOLD, Min2, and PChiB are 0.364, 0.352, 0.826, 0.504, and 0.492, respectively, when the 2nd SNP is the causal SNP. From Table 3, Supplementary Figure 5a, and Supplementary Figure 5b for *F*_*st*_ = 0.5log(2), we can see that Min2, when using all 18 SNPs, using all 18 SNPs except for the causal SNP, and using only tag SNPs, always performs much better than PChiP and SSUP, regardless of which of the 18 SNPs is the causal SNP. For example, in Table 3, the empirical powers of PChiP, SSUP, GOLD, and Min2 are 0.755, 0.795, 0.970, 0.840, and 0.875, respectively, when the 1st SNP is the causal SNP.

When the real causal SNP adopts a dominant code, we display all the results based on all 18 SNPs in Tables 4 and 5 for *F*_*st*_ = 0 and *F*_*st*_ = 0.5log(2). Other results based on 17 SNPs (excluding the causal SNP) and 5 tag SNPs are displayed in Supplementary Figure 6a, Figure 6b, Figure 7a, and Figure 7b (See Supplementary File). From these figures, we can see that Min2 performs robustly among all 5 tests over all 3 scenarios with *F*_*st*_ = 0 and 0.5log(2). For example, in Table 4, the empirical powers of PChiP, SSUP, GOLD, Min2, and PChiB are 0.598, 0.588, 0.846, 0.636, and 0.556, respectively, when the 9th SNP is the causal SNP, and the empirical powers of PChiP, SSUP, GOLD, Min2, and PChiB are 0.638, 0.382, 0.826, 0.628, and 0.496, respectively, when the 10th SNP is the causal SNP. In Table 5 for *F*_*st*_ = 0.05log(2), the empirical powers of PChiP, SSUP, GOLD, Min2, and PChiB are 0.585, 0.310, 0.786, 0.545, and 0.455, respectively, when the 11th SNP is the causal SNP.

When the real causal SNP adopts an additive code, we display all results based on all 18 SNPs in Tables 6 and 7 for *F*_*st*_ = 0 and *F*_*st*_ = 0.5log(2). Other results based on 17 SNPs (excluding the causal SNP) and 5 tag SNPs are displayed in Supplementary Figure 8a, Figure 8b, Figure 9a, and Figure 9b (See Supplementary File). From these figures, we can see that Min2 performs robustly among all 5 tests over all 3 scenarios for *F*_*st*_ = 0 and 0.5log(2). Under these 3 scenarios, the real genetic codes are additive, so it is not unexpected that the performance of PChiP is always a little better than that of Min2, regardless of which of the 18 SNPs is the causal SNP. Although SSUP sometimes has slightly better power than PChiP and Min2, it can sometimes have very low power. For example, in Table 6, the empirical powers of PChiP, SSUP, GOLD, Min2, and PChiB are 0.626, 0.402, 0.770, 0.616, and 0.400 when the 11th SNP is the causal SNP. In Table 7, for *F*_*st*_ = 0.5log(2), the empirical powers of PChiP, SSUP, GOLD, Min2, and PChiB are 0.660, 0.670, 0.800, 0.645, and 0.570, respectively, when the 9th SNP is the causal SNP.

### 2.4. The Analysis of High-Density Lipoprotein Cholesterol (HDL-C) Data from GWAS Pancreatic Cancer Data

Herein, we present an analysis of HDL-C data from GWAS pancreatic cancer data [26, 27] to illustrate our method. Plasma levels of high-density lipoprotein cholesterol are known to be heritable, but only a fraction of the heritability is explained. We developed a high-density genotyping array populated with HDL-C candidate loci selected based on the known biology of HDL metabolism, mouse genetic studies, human genetic association studies, and available GWAS data. SNP selection was based on tag SNPs but also included low-frequency nonsynonymous SNPs. We performed association analysis on the majority of reported GWAS loci (including ABCA1, CETP, GALNT2, LCAT, LIPG, LIPC, and LPL).

The data set consists of 1231 samples (case: 625 and control: 606) with 64 SNPs from the above 13 genes. Basic information about the 13 genes is presented in Supplementary Table 1 (additional file 2). We calculate the *p* values of 4 test methods, i.e., PChiP, SSUP, Min2, and PChiB, when analysing the data set. The numerical results are displayed in Table 8. From Table 8, we can see that the numerical results of Min2 are consistent with those of the other tests. For example, when investigating the association between HDL-C and gene GALNT2, including 2 SNPs, the *p* values of PChiP, SSUP, Min2, and PChiB are 0.1065, 0.1065, 0.0370, and 0.0272, respectively. For another example, when investigating the association between HDL-C and gene LPL, including 15 SNPs, the *p* values of PChiP, SSUP, Min2, and PChiB are 0.002, 0.00016, 0.002, and 0.0044, respectively. For the third example, when investigating the association between HDL-C and gene LIPG, including 2 SNPs, the *p* values of PChiP, SSUP, Min2, and PChiB are 0.0012, 0.0012, 0.0001, and 0.0002.

Because the number of SNPs in each gene is not very large in the real data, the real data do not provide a good example to illustrate the merit our test. However, this limitation does not affect our purpose of deriving a robust test. Our method focuses on the robustness in the following 3 scenarios: the genetic code for all SNPs is unknown, whether the HWE is satisfied in the original population is unknown, and a large number of SNPs exists.

## 3. Discussion

One key factor of the improved power of kernel-machine-based tests [17] and PCR is the reduced degrees of freedom. Kernel-machine-based tests make full use of possible correlations among score statistics, which is known to be advantageous for high-dimensional data [30], and are robust to the directions of association of different SNPs. Principal component analysis is a standard method of reducing the dimensionality of a large number of variables. Despite this seemingly obvious argument, the relative merits of PCR and kernel-machine-based tests remain understudied. We provide insights into the theoretical connection between kernel-machine-based tests and the PCR method. We find that when the LD extent of each pair of SNPs is somewhat strong, principal component analysis methods may have higher power than kernel-machine-based tests. PCR often has similar or higher power than kernel-machine-based tests, where the LD pattern is an important parameter for power. We will further explore the principle of selecting the number of PCs in future work.

In this work, we consider an association test between human complex diseases and genetic SNPs based on principal component analysis (PCA) since PCA is widely used in the recent literature. PCA accounts for linear combinations among SNPs. If this linearity exists, PCA is optimal. However, when how the multiple genetic SNPs influence the risk of disease is unknown, one alternative strategy is to use haplotype analysis since haplotypes can capture the LD information between markers [31–37].

We propose a novel global test (PChiB) based on the empirical Bayes score test, which is a data-adaptive linear combination of the prospective likelihood score and the retrospective likelihood score under the HWE constraint in the control population. PChiB can maintain desirable power when the real causal SNP adopts recessive and dominant codes under the HWE constraint in the control population. A small disadvantage of PChiB is that when the genetic code of the real causal SNP is additive, PChiB does not have desirable power because of the large degrees of freedom. Thus, we propose a robust test (Min2) that maintains the power gain under deviations from HWE observed in real settings, regardless of which genetic code the real causal SNP adopts. Min2 gains power by effectively using the LD among all the tested SNPs over all scenarios. Because PChiP is based on the assumption that all SNPs adopt an additive code, while PChiB and Min2 are based on the assumption that all SNPs adopt a codominant code, PChiP has low degrees of freedom and performs best when the causal SNP adopts an additive code. PChiB and Min2 may have less power than PchiP in this scenario. When the causal SNP adopts dominant or recessive codes, Min2 has desirable power, regardless of whether HWE is satisfied in the control population. We propose to use our new test Min2 for the association analysis of multilocus genotypes and complex diseases.

We propose the robust test Min2, where the *p* values are obtained via permutation and compared it with PChiB (empirical score based on all SNPs adopting codominant codes), PChiP (prospective score based on all SNPs adopting additive codes), and SSUP (a VC method based on the prospective score and all SNPs adopting an additive code). The main purpose of this article is to introduce the proposed test Min2, not to compare it with other existing tests for GWAS.

Notably, it would be a good idea to extend the proposed tests to include covariate adjustments in the logistic models. The derivation will be very complex and requires additional research. We will consider this problem in our future work. In simulations, we need to set a large sample size *n* as the number of MAF is low, so we have not considered rare variants. We may investigate the robustness about PChiB when the number of MAF is low in our further work.

## 4. Methods

### 4.1. A New Principal Chi-Squared Test

Suppose there are *n*_1_ case samples and *n*_0_ control samples and denote *n* = *n*_1_ + *n*_0_. For the *i*th (*i* = 1, ⋯, *n*) sample and *k*th (*k* = 1, ⋯, *q*) SNP, denote *G*_*ik*_ as the additive code, namely, the numbers of minor alleles taking values 0, 1, and 2. For the *i*th (*i* = 1, ⋯, *n*) sample and *k*th (*k* = 1, ⋯, *q*) SNP, denote *m*(*G*_*ik*_) as the codominant code, namely, *m*(*G*_*ik*_) = (*m*_1_(*G*_*ik*_), *m*_2_(*G*_*ik*_)) = (*I*[*G*_*ik*_ = 1], *I*[*G*_*ik*_ = 2]), where *I*[·] is an indicator function. Clearly, *m*(0) = (0, 0), *m*(1) = (1, 0), and *m*(2) = (0, 1).

For *k* = 1, ⋯, *q*, denote ${\hat{f}}_{k}$ as the estimated minor allele frequency (MAF) for the *k*th SNP in the pooled case-control sample and denote *g*_*k*_ as the number of minor allele in a genotype for the *k*th SNP in a population with values 0, 1, and 2. For *k* = 1, ⋯, *q*, denote $P_{{\hat{f}}_{k}}\left(g_{k}\right)$ as the estimated genotype frequency for the *k*th SNP. We can then obtain ${\hat{f}}_{k}=\sum_{i=1}^{n}\left\{I\left[G_{ik}=1\right]+2I\left[G_{ik}=2\right]\right\}/\left(2n\right)$, $P_{{\hat{f}}_{k}}\left(g_{k}=0\right)={\left(1-{\hat{f}}_{k}\right)}^{2}$, $P_{{\hat{f}}_{k}}\left(g_{k}=1\right)=2{\hat{f}}_{k}\left(1-{\hat{f}}_{k}\right)$, and $P_{{\hat{f}}_{k}}\left(g_{k}=2\right)={\left({\hat{f}}_{k}\right)}^{2}$. For *k* = 1, ⋯, *q*, denote a 2-dimensional row vector by $\tau_{\left(k\right)}=\left(\tau_{1k},\tau_{2k}\right)=E_{\text{HWE},{\hat{f}}_{k}}\left[m\left(g_{k}\right)\right]-\bar{m}\left(g_{k}\right)=\left(E_{\text{HWE},{\hat{f}}_{k}}\left[m_{1}\left(g_{k}\right)\right]-{\bar{m}}_{1}\left(g_{k}\right),E_{\text{HWE},{\hat{f}}_{k}}\left[m_{2}\left(g_{k}\right)\right]-{\bar{m}}_{2}\left(g_{k}\right)\right)$, where $E_{\text{HWE},{\hat{f}}_{k}}\left[m\left(g_{k}\right)\right]=\sum_{g_{k}=0,1,2}m\left(g_{k}\right)P_{{\hat{f}}_{k}}\left(g_{k}\right)=\left(\sum_{g_{k}=0,1,2}m_{1}\left(g_{k}\right)P_{{\hat{f}}_{k}}\left(g_{k}\right),\sum_{g_{k}=0,1,2}m_{2}\left(g_{k}\right)P_{{\hat{f}}_{k}}\left(g_{k}\right)\right)$ is the expected value of *m*(*g*_*k*_) under HWE, and $\bar{m}\left(g_{k}\right)=\left({\bar{m}}_{1}\left(g_{k}\right),{\bar{m}}_{2}\left(g_{k}\right)\right)$ is the pooled sample mean of *m*(*g*_*k*_) = (*m*_1_(*g*_*k*_), *m*_2_(*g*_*k*_)), namely, $\bar{m}\left(g_{k}\right)=\sum_{i=1}^{n}m\left(G_{ik}\right)/n=\left(\sum_{i=1}^{n}m_{1}\left(G_{ik}\right)/n,\sum_{i=1}^{n}m_{2}\left(G_{ik}\right)/n\right)$. For *k* = 1, ⋯, *q*, denote $s_{{\bar{m}}_{1}\left(g_{k}\right)}^{2}$ as the pooled sample variance of *m*_1_(*g*_*k*_), namely, the variance of *m*_1_(*G*_1*k*_), ⋯, *m*_1_(*G*_*nk*_) and denote $s_{{\bar{m}}_{2}\left(g_{k}\right)}^{2}$ as the pooled sample variance of *m*_2_(*g*_*k*_), namely, the variance of *m*_2_(*G*_1*k*_), ⋯, *m*_2_(*G*_*nk*_). For *k* = 1, ⋯, *q*, denote a diagonal matrix *W*_*k*_ with elements equal to $\left(s_{{\bar{m}}_{1}\left(g_{k}\right)}^{2}/n\right)/\left(\left(s_{{\bar{m}}_{1}\left(g_{k}\right)}^{2}\right)/n+\tau_{1k}^{2}\right)$ and $\left(s_{{\bar{m}}_{2}\left(g_{k}\right)}^{2}/n\right)/\left(\left(s_{{\bar{m}}_{2}\left(g_{k}\right)}^{2}\right)/n+\tau_{2k}^{2}\right)$. Clearly, *W*_*k*_ is extended from the weight proposed by Luo et al. [25] and Chatterjee et al. [38] when an additive (dominant or recessive) code is adopted. The weight matrix *W*_*k*_ is data adaptive. When codominant coding is adopted, by means of *W*_*k*_, we propose the empirical Bayes score for the *k*th (*k* = 1, ⋯, *q*) SNP with the following form:

(3) $$\begin{array}{c} U_{B,k}=\overset{n_{1}}{\underset{i=1}{\sum }}\left\{m\left(G_{ik}\right)-\left[E_{\text{HWE},{\hat{f}}_{k}}\left[m\left(g_{k}\right)\right]W_{k}+\bar{m}\left(g_{k}\right)\left(I_{2\times 2}-W_{k}\right)\right]\right\}, \end{array}$$

where *I*_2×2_ is an identity matrix with dimension 2.

Let *U*_(*B*)_ denote the vector of empirical Bayes scores for all *q* SNPs, namely, *U*_(*B*)_ = (*U*_*B*,1_, *U*_*B*,2_, ⋯, *U*_*B*,*q*_), which is of length 2*q*. Denote the estimated asymptotic covariance matrix by *V*_*B*_ (See Supplementary File) for empirical Bayes score vector *U*_(*B*)_. A common test for whether all *q* markers can be jointly built, similar to the Hotelling *T*^2^ statistic, is *T*^2^ = *U*_(*B*)_*V*_*B*_^−1^*U*_(*B*)_^*T*^, where ‘*T*' indicates the transpose of a vector or matrix. Our proposed new global statistic is based on the eigenvalue decomposition of covariance matrix *V*_*B*_, as follows. For *k* = 1, 2, ⋯, 2*q*, denote *λ*_*k*_ and *ξ*_*k*_ (a 2*q* × 1 column vector) as the eigenvalue and corresponding eigenvector of covariance matrix *V*_*B*_. Let *λ* = (*λ*_1_, *λ*_2_, ⋯, *λ*_2*q*_) and *ξ* = (*ξ*_1_, *ξ*_2_, ⋯, *ξ*_2*q*_) denote the eigenvalues and corresponding eigenvectors of covariance matrix *V*_*B*_. We then have *ξ*^*T*^*V*_*B*_*ξ* = diag(*λ*_1_, *λ*_2_, ⋯, *λ*_2*q*_), and *V*_*B*_ can be written as *ξ*diag(*λ*_1_, *λ*_2_, ⋯, *λ*_2*q*_)*ξ*^*T*^. Since the norm of the eigenvector is unity and *V*_*B*_^−1^ can be written as *ξ*diag(*λ*_1_^−1^, *λ*_2_^−1^, ⋯, *λ*_2*q*_^−1^)*ξ*^*T*^, the test statistic *T*^2^ can be written as

(4) $$\begin{array}{c} T^{2}=\left(U_{\left(B\right)}\xi \right)\mathrm{diag}\left(\lambda_{1}^{-1},\lambda_{2}^{-1},\cdots ,\lambda_{2q}^{-1}\right)\left(\xi^{T}U_{\left(B\right)}^{T}\right)=\overset{2q}{\underset{k=1}{\sum }}{\left(U_{\left(B\right)}\xi_{k}\right)}^{2}/\lambda_{k}. \end{array}$$

Note that *U*_(*B*)_*ξ*_*k*_, is a linear combination of the score for each individual SNP *U*_*B*,*k*_ with var[*U*_(*B*)_*ξ*_*k*_] = *λ*_*k*_ for *k* = 1, 2, ⋯, 2*q*. We propose to utilize the first *s*(1 ≤ *s* ≤ 2*q*) summands in *T*^2^ to test the null hypothesis and denote the resultant test statistic as follows:

(5) $$\begin{array}{c} \text{PChiB}=\overset{s}{\underset{k=1}{\sum }}\frac{{\left(U_{\left(B\right)}\xi_{k}\right)}^{2}}{\lambda_{k}}. \end{array}$$

Due to the orthogonality of *ξ*_1_, ⋯, *ξ*_*s*_, (*U*_(*B*)_*ξ*_1_)^2^/*λ*_1_, ⋯, (*U*_(*B*)_*ξ*_*s*_)^2^/*λ*_*s*_ are independent. Because (*U*_(*B*)_*ξ*_1_)^2^/*λ*_1_, ⋯, (*U*_(*B*)_*ξ*_*s*_)^2^/*λ*_*s*_ are all asymptotically normally distributed with mean 0 and variance 1 under the null hypothesis that the genomic region spanned by the *q* SNPs is not associated with the phenotype status of interest, PChiB is asymptotically distributed as a Chi-squared variable with *s* degrees of freedom under the null hypothesis.

A remaining issue is how to select the number of summands *s*. Note that PChiB is based on eigenvalue decomposition, similar to the standard PCR. Many criteria for selecting *s* have been introduced in the literature [39]. It has been shown that using the top principal components that explain 80~90% of the genetic variability is sufficient [19, 20, 23]. We select *s* according to the same principal, i.e., that the top *s* principal components can explain approximately 85% of the genetic variability. This strategy is supported by the connection between PChiB and PCR (see the next subsection). In fact, the number of principal components affects the power of the principal component test [40]. When the LD extent of each pair of SNPs is very strong, the top one principal component alone has desirable power. When the LD extent of each pair of SNPs is somewhat strong, using the top principal components that explain 80~90% of the genetic variability is a robust method.

### 4.2. Understanding PChiB through an Exposition of PCR

We revisit PChiB based on only the standard prospective likelihood score under additive coding for *k*th (*k* = 1, ⋯, *q*) and establish its equivalence to PCR [19, 20]. This equivalence sheds light on the promise of increased power of PChiB since PCR has been established to be a promising method for multi-SNP association analysis. In PCR, the phenotype variable is regressed on only a few of the top principal components (PCs) that summarize approximately 80-90% of the genetic variability. The PCs represent the directions in which most of the variability in the data occurs, as identified by the eigenvalue decomposition of the variance-covariance matrix of the centred raw genotype scores. Each principal component is a linear combination of genotype scores for all SNPs, and all principal components are uncorrelated with each other.

Here, we present the standard prospective likelihood score under additive genetic coding. The collection of all *q* prospective score functions, denoted by ${\tilde{U}}_{\left(P\right)}=\left({\tilde{U}}_{P,1},{\tilde{U}}_{P,2},\cdots ,{\tilde{U}}_{P,q}\right)$, is asymptotically distributed as multivariate normal with mean (0,⋯,0)_*q*×1_ and variance-covariance matrix ${\tilde{V}}_{P}$ under the null hypothesis. Let *Y* = (*Y*_1_, ⋯, *Y*_*n*_)^*T*^, $\bar{Y}=\sum_{i=1}^{n}Y_{i}/n$. For *k* = 1, 2, ⋯, *q*, let ${\bar{G}}_{k}=\sum_{i=1}^{n}G_{ik}/n$ and $\bar{G}=\left({\bar{G}}_{1},\cdots ,{\bar{G}}_{q}\right)$. For *i* = 1, 2, ⋯, *n*, let *G*_(*i*)_ = (*G*_*i*1_, ⋯, *G*_*iq*_)^*T*^. Denote *G* as a genotype matrix with *i*th row and *k*th column element *G*_*ik*_ for *i* = 1, ⋯, *n*,and *k* = 1, ⋯, *q*. Let $\bar{1}$ be a column vector with all elements 1 and length *n*. In matrix form, ${\tilde{U}}_{\left(P\right)}^{T}=\sum_{i=1}^{n}\left(Y_{i}-\bar{Y}\right)G_{\left(i\right)}=G^{T}\left(Y-\bar{Y}\bar{1}\right)$, and its covariance matrix ${\tilde{V}}_{P}=\bar{Y}\left(1-\bar{Y}\right)\sum_{i=1}^{n}\left[G_{\left(i\right)}-\bar{G}\right]{\left[G_{\left(i\right)}-\bar{G}\right]}^{T}$. Now, let *A* = [*a*_1_, *a*_2_, ⋯, *a*_*q*_] be a *q* × *q* matrix whose *k*th column is the characteristic vector of the matrix ${\tilde{V}}_{P}\left(k=1,\cdots ,q\right)$, and let ${\tilde{\lambda }}_{1}\geq {\tilde{\lambda }}_{2}\geq \cdots \geq {\tilde{\lambda }}_{q}$ be its eigenvalues. Denote orthogonal transformation $\tilde{G}=GA$. The likelihood score based on a logistic regression of *Y* on $\tilde{G}$ is ${\tilde{U}}_{\left(P\right)}={\tilde{G}}^{T}\left(Y-\bar{Y}\bar{1}\right)$. The covariance matrix of ${\tilde{U}}_{\left(P\right)}$ is a diagonal matrix with elements ${\tilde{\lambda }}_{1},{\tilde{\lambda }}_{2},\cdots ,{\tilde{\lambda }}_{q}.$

Suppose that we consider the first $\tilde{s}$($1\leq \tilde{s}\leq q$) PCs as follows. Let $A_{\tilde{s}}=\left[a_{1},a_{2},\cdots ,a_{\tilde{s}}\right]$ be a $q\times \tilde{s}$ matrix containing the first $\tilde{s}$ eigenvectors, and let ${\tilde{G}}_{\tilde{s}}=GA_{\tilde{s}}$. The standard PCA test based on the score statistic for testing the association between *Y* and ${\tilde{G}}_{\tilde{s}}$ from the logistic regression model is exactly equal to ${\left(Y-\bar{Y}\bar{1}\right)}^{T}GA_{\tilde{s}}\mathrm{diag}\left({\tilde{\lambda }}_{1}^{-1},{\tilde{\lambda }}_{2}^{-1},\cdots ,{\tilde{\lambda }}_{\tilde{s}}^{-1}\right)A_{\tilde{s}}^{T}G^{T}\left(Y-\bar{Y}\bar{1}\right)$, which is denoted by *PChiP*, and is the same as our proposed method when the adopted genetic code is additive code. Denote ${\tilde{T}}_{k}={\left(Y-\bar{Y}\bar{1}\right)}^{T}Ga_{k}a_{k}^{T}G^{T}\left(Y-\bar{Y}\bar{1}\right)/\lambda_{k}$, *k* = 1, 2, ⋯, *q*. When adopting additive code, the standard Hotelling *T*^2^ statistic is equal to $\sum_{k=1}^{q}{\tilde{T}}_{k}$, and the PChiP statistic reduces to $\sum_{k=1}^{\tilde{s}}{\tilde{T}}_{k}$.

The proposed statistic (in this situation, equivalent to PCR) can be shown to be closely related to a statistic called the sum of squared score test based on prospective likelihood [12], which is denoted by SSUP. SSUP is obtained as SSUP$={\tilde{U}}_{\left(P\right)}{\tilde{U}}_{\left(P\right)}^{T}=\sum_{j=1}^{q}{\tilde{U}}_{P,k}^{2}$, and it can be expressed as SSUP$=\sum_{j=1}^{q}{\tilde{\lambda }}_{k}\left[{\left({\tilde{U}}_{\left(P\right)}a_{k}^{T}\right)}^{2}/{\tilde{\lambda }}_{k}\right]$. Therefore, *S*SUP and PChiB use different weights for the contributions of the PCs: SSUP weights all PCs by the eigenvalues, whereas PChiB assigns equal weights to the top PCs. SSUP allows PCs with small eigenvalues to make additional contributions to the test, but PChiB discards PCs with small eigenvalues to reduce the degrees of freedom. This difference has implications on their relative power, which depends critically on the structure of variance-covariance matrix and, therefore, the LD structure of the assessed genomic region.

## Figures and Tables

**Table 1:** Size and haplotypes with frequencies for gene NAT2.

Haplotype	Frequency
443423442114244211	0.279
214242244112422433	0.246
413443444332224231	0.211
214242224112422433	0.092
214243444332222431	0.042
413243444112422233	0.025
413443444332244231	0.018
443423444332224231	0.017
214242244112224233	0.017
413423442134244211	0.011
244242244112422433	0.008
413243224112422433	0.008
413443442332422433	0.008
214242224132422433	0.008
413423422134244211	0.006
214242244132422433	0.002

**Table 2:** Empirical powers (based on all 18 SNPs) of GOLD, PChiP, SSUP, PChiB, and Min2. Each SNP is treated as the causal locus in turn, which has a recessive effect, with simulated odds ratio 1.5 and *F*_*st*_t = 0 based on 1000 controls, 1000 cases, and 500 iterations.

Causal SNP no.	PChiP	SSUP	GOLD	Min2	PChiB
1	0.672	0.738	0.948	0.764	0.764
2	0.364	0.352	0.826	0.504	0.492
3	0.678	0.768	0.954	0.784	0.796
4	0.748	0.826	0.972	0.846	0.842
5	0.428	0.394	0.816	0.546	0.534
6	0.642	0.704	0.926	0.726	0.732
7	0.588	0.638	0.932	0.736	0.73
8	0.048	0.042	0.186	0.054	0.024
9	0.42	0.366	0.81	0.506	0.524
10	0.348	0.168	0.778	0.372	0.286
11	0.398	0.186	0.844	0.378	0.2
12	0.434	0.4	0.812	0.554	0.542
13	0.586	0.642	0.938	0.684	0.708
14	0.428	0.426	0.836	0.54	0.522
15	0.73	0.818	0.978	0.822	0.826
16	0.678	0.746	0.972	0.78	0.808
17	0.34	0.328	0.778	0.51	0.518
18	0.71	0.768	0.954	0.802	0.794

**Table 3:** Empirical powers (based on all 18 SNPs) of GOLD, PChiP, SSUP, PChiB, and Min2. Each SNP is treated as the causal locus in turn, which has a recessive effect, with simulated odd ratios 1.5 and *F*_*st*_ = 0.5log(2.0) based on 1000 controls, 1000 cases, and 500 iterations.

Causal SNP no.	PChiP	SSUP	GOLD	Min2	PChiB
1	0.755	0.795	0.97	0.84	0.875
2	0.45	0.46	0.865	0.51	0.605
3	0.765	0.835	0.965	0.855	0.885
4	0.835	0.925	0.99	0.84	0.88
5	0.555	0.58	0.85	0.605	0.67
6	0.69	0.77	0.935	0.785	0.765
7	0.65	0.715	0.965	0.74	0.79
8	0.06	0.085	0.37	0.07	0.1
9	0.515	0.48	0.905	0.65	0.755
10	0.535	0.28	0.825	0.6	0.59
11	0.58	0.33	0.88	0.625	0.665
12	0.48	0.475	0.83	0.62	0.665
13	0.695	0.765	0.94	0.735	0.79
14	0.58	0.595	0.895	0.655	0.7
15	0.79	0.875	0.98	0.84	0.88
16	0.725	0.805	0.97	0.805	0.865
17	0.52	0.495	0.83	0.61	0.65
18	0.785	0.825	0.955	0.86	0.875

**Table 4:** Empirical powers (based on all 18 SNPs) of GOLD, PChiP, SSUP, PChiB, and Min2. Each SNP is treated as the causal locus in turn, which has a dominant effect, with simulated odds ratio 1.3 and *F*_*st*_ = 0 based on 1000 controls, 1000 cases, and 500 iterations.

Causal SNP no.	PChiP	SSUP	GOLD	Min2	PChiB
1	0.51	0.56	0.76	0.532	0.456
2	0.57	0.552	0.822	0.564	0.49
3	0.486	0.576	0.79	0.532	0.438
4	0.448	0.532	0.74	0.476	0.416
5	0.644	0.626	0.824	0.628	0.518
6	0.556	0.61	0.808	0.576	0.516
7	0.568	0.63	0.79	0.596	0.504
8	0.13	0.152	0.712	0.128	0.078
9	0.598	0.588	0.846	0.636	0.556
10	0.638	0.382	0.826	0.628	0.496
11	0.574	0.338	0.818	0.586	0.51
12	0.614	0.614	0.836	0.622	0.56
13	0.506	0.58	0.79	0.548	0.502
14	0.584	0.578	0.836	0.576	0.51
15	0.458	0.518	0.756	0.482	0.388
16	0.462	0.538	0.808	0.478	0.418
17	0.694	0.662	0.822	0.676	0.598
18	0.492	0.55	0.76	0.51	0.448

**Table 5:** Empirical powers (based on all 18 SNPs) of GOLD, PChiP, SSUP, PChiB, and Min2. Each SNP is treated as the causal locus in turn, which has a dominant effect, with simulated odd ratio 1.3 and *F*_*st*_ = 0.5log(2.0) based on 1000 controls, 1000 cases, and 500 iterations.

Causal SNP no.	PChiP	SSUP	GOLD	Min2	PChiB
1	0.56	0.645	0.774	0.47	0.45
2	0.55	0.505	0.816	0.475	0.455
3	0.5	0.55	0.778	0.44	0.445
4	0.455	0.5	0.75	0.4	0.43
5	0.615	0.645	0.834	0.54	0.51
6	0.62	0.68	0.816	0.565	0.61
7	0.56	0.61	0.812	0.46	0.515
8	0.15	0.19	0.712	0.12	0.145
9	0.58	0.56	0.834	0.53	0.515
10	0.67	0.435	0.822	0.6	0.56
11	0.585	0.31	0.786	0.545	0.455
12	0.61	0.6	0.852	0.57	0.555
13	0.485	0.575	0.812	0.445	0.455
14	0.68	0.645	0.804	0.56	0.53
15	0.505	0.545	0.776	0.415	0.43
16	0.455	0.55	0.79	0.43	0.44
17	0.66	0.64	0.826	0.61	0.6
18	0.51	0.55	0.76	0.475	0.46

**Table 6:** Empirical powers (based on all 18 SNPs) of GOLD, PChiP, SSUP, PChiB, and Min2. Each SNP is treated as the causal locus in turn, which has an additive effect, with simulated odds ratio 1.2 and *F*_*st*_ = 0 based on 1000 controls, 1000 cases, and 500 iterations.

Causal SNP no.	PChiP	SSUP	GOLD	Min2	PChiB
1	0.694	0.748	0.798	0.644	0.466
2	0.614	0.584	0.784	0.594	0.404
3	0.654	0.728	0.818	0.614	0.424
4	0.706	0.78	0.8	0.678	0.482
5	0.666	0.656	0.798	0.626	0.428
6	0.654	0.736	0.796	0.618	0.462
7	0.724	0.77	0.814	0.702	0.484
8	0.102	0.114	0.504	0.09	0.052
9	0.632	0.63	0.76	0.594	0.408
10	0.644	0.36	0.77	0.614	0.352
11	0.626	0.402	0.77	0.616	0.4
12	0.674	0.652	0.774	0.606	0.432
13	0.708	0.768	0.802	0.682	0.468
14	0.644	0.618	0.804	0.604	0.422
15	0.678	0.782	0.816	0.656	0.484
16	0.632	0.71	0.794	0.612	0.428
17	0.696	0.662	0.754	0.652	0.444
18	0.688	0.756	0.798	0.652	0.454

**Table 7:** Empirical powers (based on all 18 SNPs) of GOLD, PChiP, SSUP, PChiB, and Min2. Each SNP is treated as the causal locus in turn, which has an additive effect, with simulated odds ratio 1.2 and *F*_*st*_ = 0.5log(2.0) based on 1000 controls, 1000 cases, and 500 iterations.

Causal SNP no.	PChiP	SSUP	GOLD	Min2	PChiB
1	0.76	0.805	0.855	0.705	0.625
2	0.66	0.6	0.795	0.585	0.55
3	0.745	0.78	0.825	0.725	0.655
4	0.765	0.84	0.86	0.735	0.695
5	0.755	0.72	0.825	0.69	0.57
6	0.77	0.795	0.825	0.685	0.61
7	0.725	0.765	0.84	0.665	0.625
8	0.155	0.19	0.585	0.145	0.14
9	0.66	0.67	0.8	0.645	0.57
10	0.725	0.53	0.82	0.69	0.565
11	0.665	0.435	0.835	0.63	0.51
12	0.73	0.695	0.82	0.62	0.59
13	0.695	0.74	0.85	0.675	0.6
14	0.7	0.67	0.81	0.655	0.55
15	0.66	0.725	0.82	0.635	0.545
16	0.635	0.725	0.82	0.58	0.535
17	0.72	0.705	0.81	0.705	0.57
18	0.72	0.76	0.845	0.695	0.62

**Table 8:** *p* values of tests PChiP, SSUP, Min2, and PChiB when analysing 7 genes.

Gene	SNP nos.	PChiP	SSUP	Min2	PChiB
GALNT2	2	0.1065	0.1065	0.0370	0.0272
LPL	15	0.0020	0.00016	0.0020	0.0044
ABCA1	3	0.0311	0.0121	0.040	0.0782
LIPC	9	0.0069	0.0019	0.0050	0.0669
CETP	25	6.051e-13	3.278e-13	7.615e-14	1.114e-16
LCAT	2	0.9981	0.9999	0.9700	0.9297
LIPG	2	0.0012	0.0012	0.0001	0.0002

## Data Availability

Data available on request.

## Supplementary Materials

Supplementary Materials Derivation of the asymptotic variance estimate for EB score test UB. 2. Supplementary tables and figures for Results.
