# Supplementary material for: Statistical Method Based on Bayes-Type Empirical Score Test for Assessing Genetic Association with Multilocus Genotype Data
Source: Int J Genomics. 2020 May 6;2020:4708152. doi: 10.1155/2020/4708152 (PMC7229558; doi:10.1155/2020/4708152)
Supplement: Supplementary Materials — Derivation of the asymptotic variance estimate for EB score test UB. 2. Supplementary tables and figures for Results. [file 4708152.f1.pdf]

Supplementary Materials for

Statistical Method Based on Bayes-type Empirical Score Test for Assessing Genetic Association with Multilocus Genotype Data

Yi Tian[1], Li Ma[2], Xiaohong Cai[2], and Jiayan Zhu[2,*]

[1] School of Mathematics and Statistics, Central China Normal University, Wuhan, 430079, China

[2] School of Information Engineering, Hubei University of Chinese Medicine, Wuhan, 430065, China

[*] Corresponding author: zhujiayan999@163.com

Contents

1. Derivation of the asymptotic variance estimate for EB score test U_B.

2. Supplementary Tables and Figures for "Results" section.

1. Derivation of the asymptotic variance estimate for EB score test U_B.

Let $U_{P,k}$ and $U_{R,k}$ denote the prospective and retrospective likelihood score functions, respectively for $k = 1, \cdots, q$. We can get,
$U_{P,k} = \frac{n_1 n_0}{n_1+n_0}\left[\frac{1}{n_1}\sum_{i=1}^{n_1} m(G_{ik}) - \frac{1}{n_0}\sum_{j=1}^{n_0} m(G_{jk})\right]$, and $U_{R,k} = \sum_{i=1}^{n_1}\left[m(G_{ik}) - E_{HWE,\hat{f}_k}[m(g_k)]\right]$,
for $k = 1, \cdots, q$.

The empirical Bayes score can be expressed as $U_{B,k} = U_{P,k}(I_{2\times 2} - W_k) + U_{R,k}W_k$. We show that the asymptotic variance of $U_{B,k}$ can be based on the following "influence function" $\tilde{U}_{B,k}$:

$$\tilde{U}_{B,k} = U_{P,k}(I_{2\times 2} - W_k) + U_{R,k}W_k$$
$$= \frac{n_1 n_0}{n}\left[\frac{1}{n_1}\sum_{i=1}^{n_1} m(G_{ik}) - \frac{1}{n_0}\sum_{j=1}^{n_0} m(G_{jk})\right](I_{2\times 2} - W_k)$$
$$+\left\{\sum_{i=1}^{n_1}\left[m(G_{ik}) - E_{HWE,\hat{f}_k}[m(g_k)]\right] - \frac{n_1}{2n}c(f_k)\sum_{i=1}^{n}\left[I(G_{ik}=1) + 2I(G_{ik}=2) - 2f_k\right]\right\}W_k,$$

where $c(f_k) = \sum_{g_k=0,1,2}\left[m(g_k)\times\frac{g_k - 2f_k}{f_k(1-f_k)}p_{0g}(f_k)\right]$, $p_{00}(f_k) = (1-f_k)^2$, $p_{01}(f_k) = 2f_k(1-f_k)$ and $p_{02}(f_k) = f_k^2$. The rearrangement of the above terms is as follows:

$$\sum_{i=1}^{n_1}\left\{\frac{n_0}{n}m(G_{ik})(I_{2\times 2} - W_k) + \left[m(G_{ik}) - E_{HWE,f_k}[m(g_k)]\right]W_k\right.$$
$$\left.-\frac{n_1 W_k}{2n}\left[I(G_{ik}=1) + 2I(G_{ik}=2) - 2f_k\right]c(f_k)W_k\right\}$$
$$-\sum_{i=n_1+1}^{n}\left\{\frac{n_1}{n}m(G_{ik})(I_{2\times 2} - W_k) + \frac{n_1}{2n}\left[I(G_{ik}=1) + 2I(G_{ik}=2) - 2f_k\right]c(f_k)W_k\right\}.$$

Our actual calculation for estimating the covariance matrix V_B was based on the covariance of the above $n \times 2q$ rearranged data.

2. Supplementary Tables and Figures

Supplementary Table1 presents brief information about 64 markers from 13 genes in real data analysis. Supplementary Figure1 graphically illustrates LD structures plot for gene NAT2 by using HAPLOVIEW software. Supplementary Figure2a-Figure9b graphically illustrates more simulation results about 5 test.

Supplementary Table 1. Brief information about 64 markers from 13 genes(1) (Source:GWAS(1) and IBC(2)).

	CHR	SNP	BP.position	Locus	Source
1	1	rs2144300	228361539	GALNT2	1
2	1	rs4846914	228362314	GALNT2	1
3	8	rs3779788	19847373	LPL	2
4	8	rs255	19856181	LPL	2
5	8	rs256	19856247	LPL	2
6	8	rs263	19857092	LPL	2
7	8	rs264	19857460	LPL	2
8	8	rs271	19857982	LPL	2
9	8	rs301	19861214	LPL	2
10	8	rs328	19864004	LPL	2
11	8	rs331	19864685	LPL	2
12	8	rs12679834	19864713	LPL	2
13	8	rs3208305	19867928	LPL	2
14	8	rs3735964	19868325	LPL	2
15	8	rs13702	19868772	LPL	2
16	8	rs3916027	19869148	LPL	2
17	8	rs2197089	19870653	LPL	1
18	9	rs3890182	106687476	ABCA1	1
19	9	rs2275544	106691033	ABCA1	1
20	9	rs1883025	106704122	ABCA1	1

Supplementary Table 1. Brief information about 64 markers from 13 genes(2) (Source:GWAS(1) and IBC(2)).

21	15	rs11635491	56507033	LIPC	2
22	15	rs1800588	56510967	LIPC	2
23	15	rs2070895	56511231	LIPC	2
24	15	rs8034802	56512084	LIPC	2
25	15	rs8033940	56512134	LIPC	2
26	15	rs261332	56514617	LIPC	2
27	15	rs588136	56517790	LIPC	2
28	15	rs261341	56518859	LIPC	2
29	15	rs261338	56522297	LIPC	2
30	16	rs13306677	55483696	CETP	1
31	16	rs6499861	55548996	CETP	2
32	16	rs6499863	55549518	CETP	2
33	16	rs12708967	55550712	CETP	2
34	16	rs3764261	55550825	CETP	1
35	16	rs12720918	55551713	CETP	2
36	16	rs17231506	55552029	CETP	2
37	16	rs4783961	55552395	CETP	2
38	16	rs1800775	55552737	CETP	2
39	16	rs711752	55553712	CETP	2
40	16	rs708272	55553789	CETP	2

Supplementary Table 1. Brief information about 64 markers from 13 genes(3) (Source:GWAS(1) and IBC(2)).

41	16	rs1864163	55554734	CETP	2
42	16	rs7203984	55556759	CETP	2
43	16	rs11508026	55556829	CETP	2
44	16	rs12720922	55558386	CETP	2
45	16	rs9939224	55560233	CETP	2
46	16	rs11076174	55560647	CETP	2
47	16	rs1532625	55562802	CETP	2
48	16	rs1532624	55562980	CETP	2
49	16	rs11076175	55563879	CETP	2
50	16	rs7499892	55564091	CETP	2
51	16	rs11076176	55564947	CETP	2
52	16	rs289714	55564952	CETP	2
53	16	rs5880	55572592	CETP	2
54	16	rs1800777	55574820	CETP	2
55	16	rs2292318	66543207	LCAT	1
56	16	rs255052	66582496	LCAT	1
57	18	rs1943981	45423813	LIPG	1
58	18	rs2156552	45435666	LIPG	1

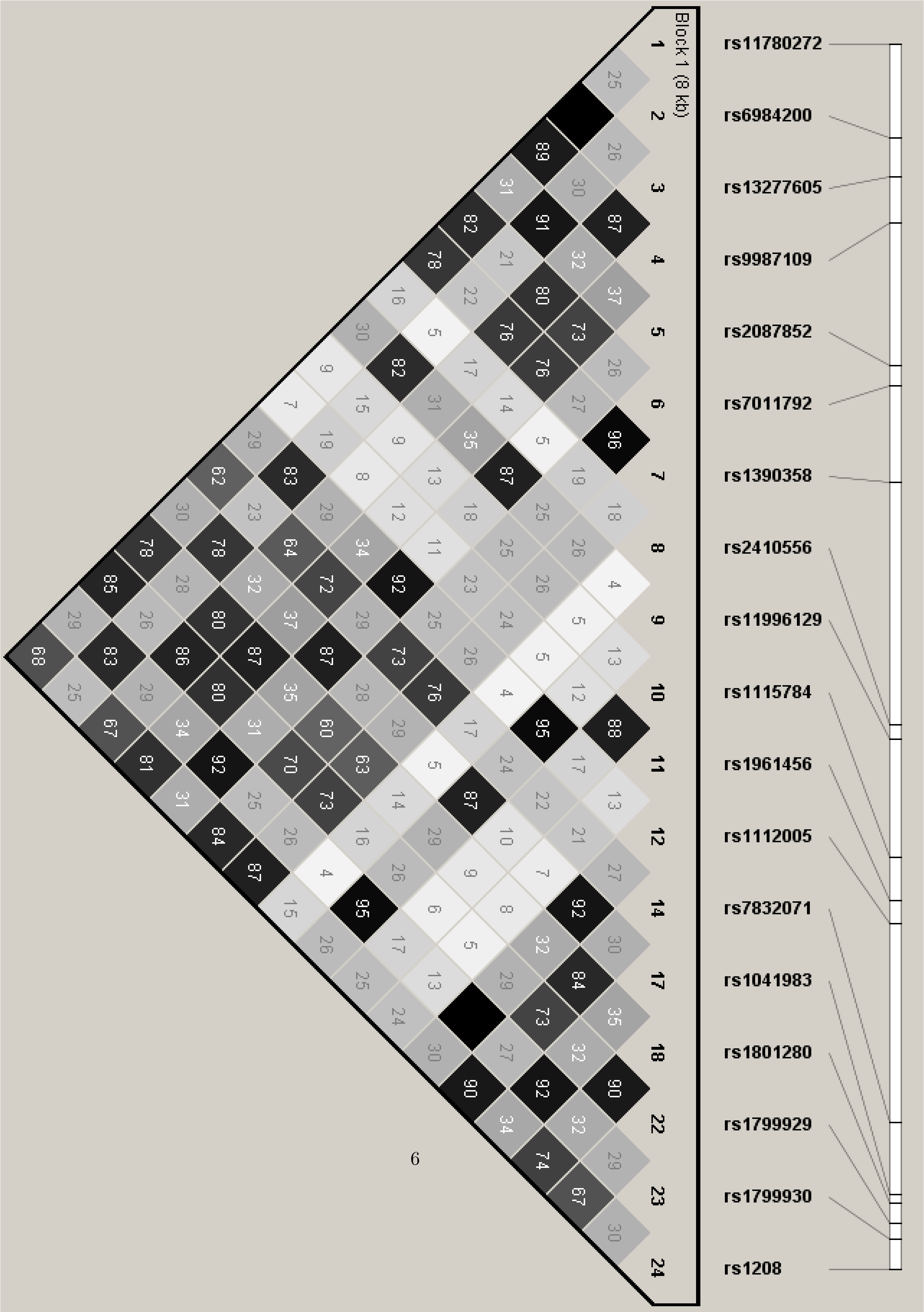

Figure 1. LD Plot of 18 SNPs within the Gene NAT2 by HAPLOVIEW software.

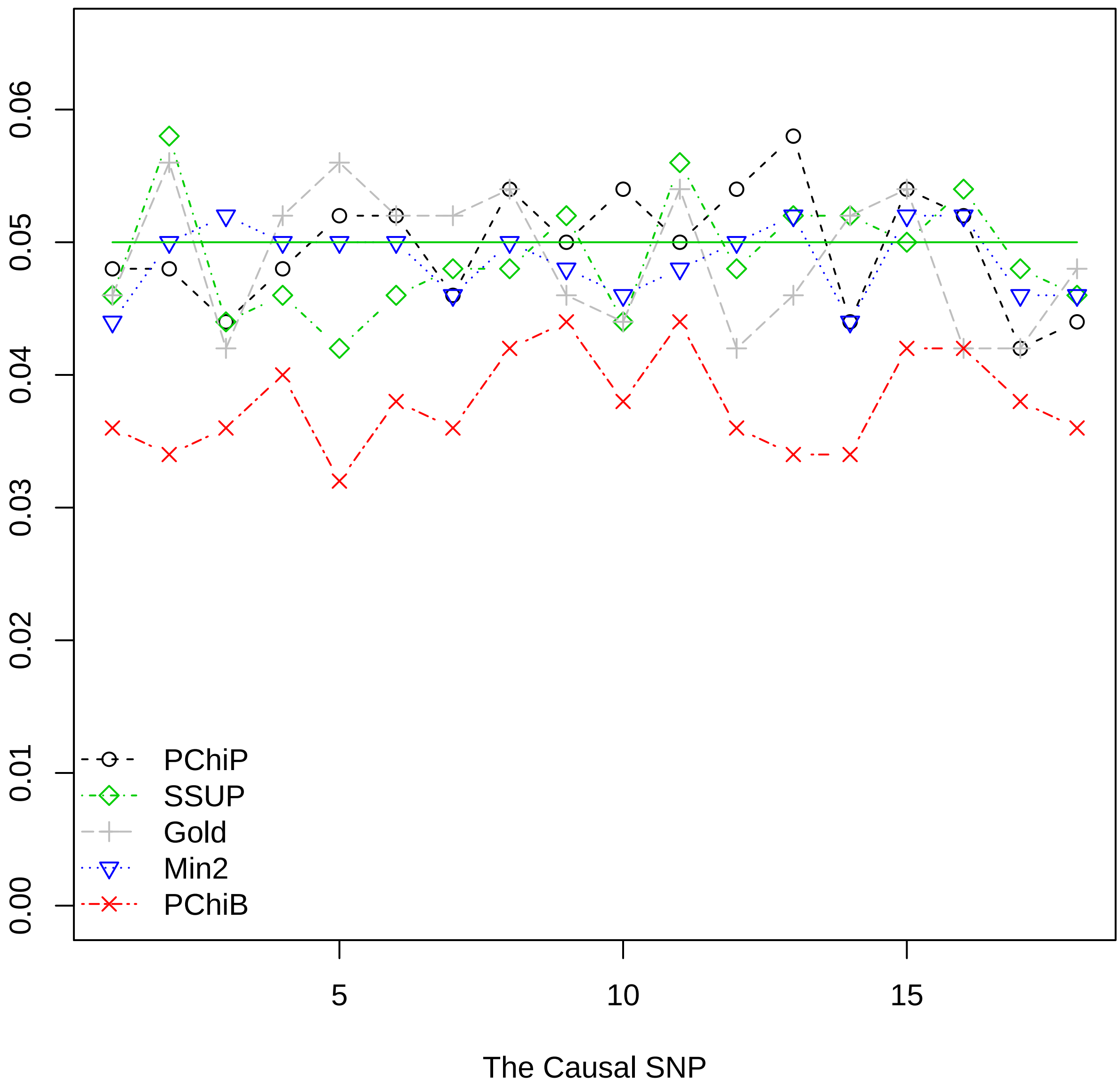

Figure 2a. Empirical null hypothesis rejection rates(based on 17 SNPs excluding the causal SNP) of GOLD, PChiP, SSUP, PChiB and Min2. Each SNP is treated as the causal locus in turn, which has a additive effect with simulated odds ratios 1.0 and Fst=0 based on 1000 controls, 1000 cases and 500 iterations.

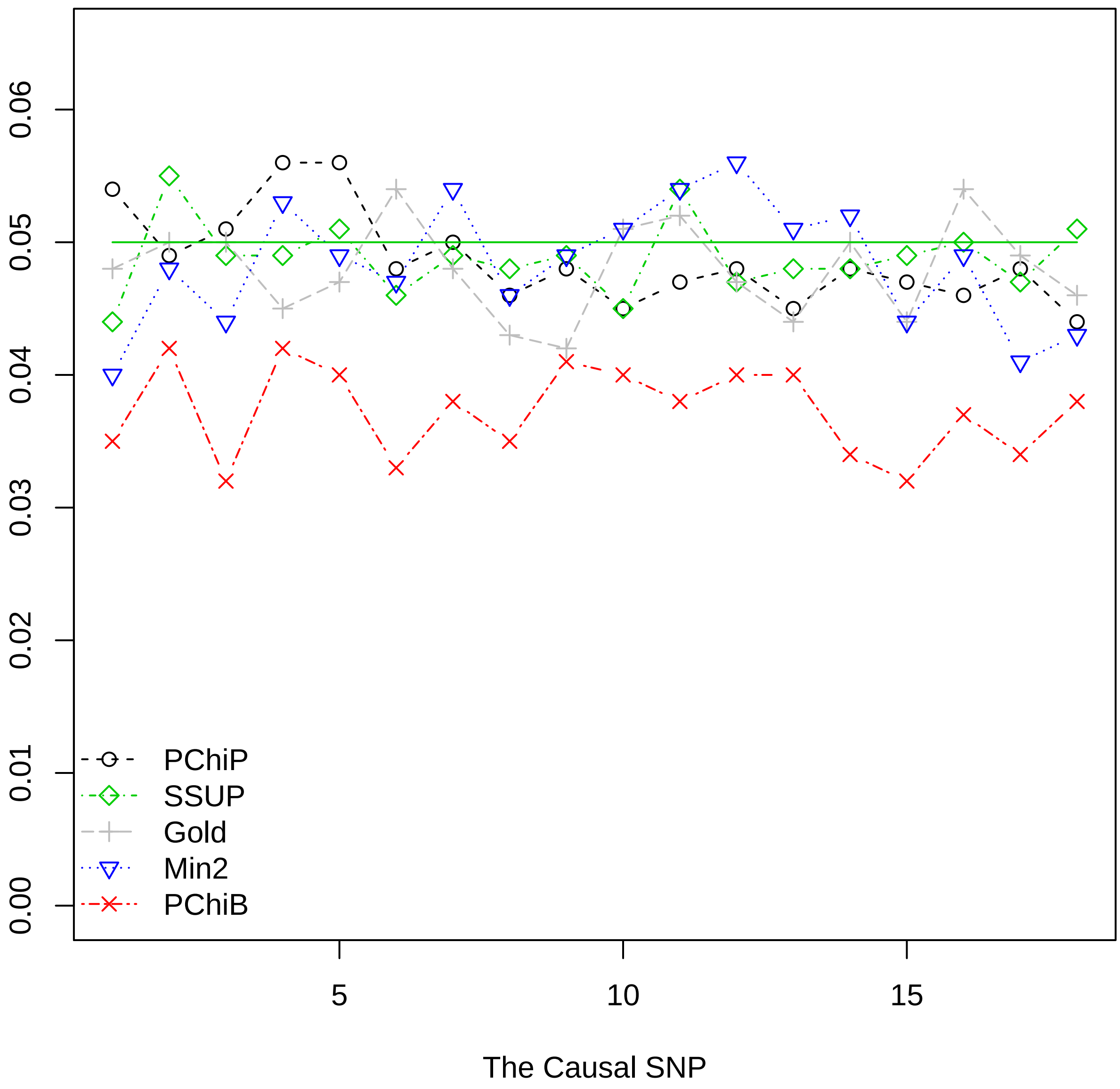

Figure 2b. Empirical null hypothesis rejection rates(based on 5 tag labeled SNPs) of GOLD, PChiP, SSUP, PChiB and Min2. Each SNP is treated as the causal locus in turn, which has a additive effect with simulated odds ratios 1.0 and Fst=0 based on 1000 controls, 1000 cases and 500 iterations.

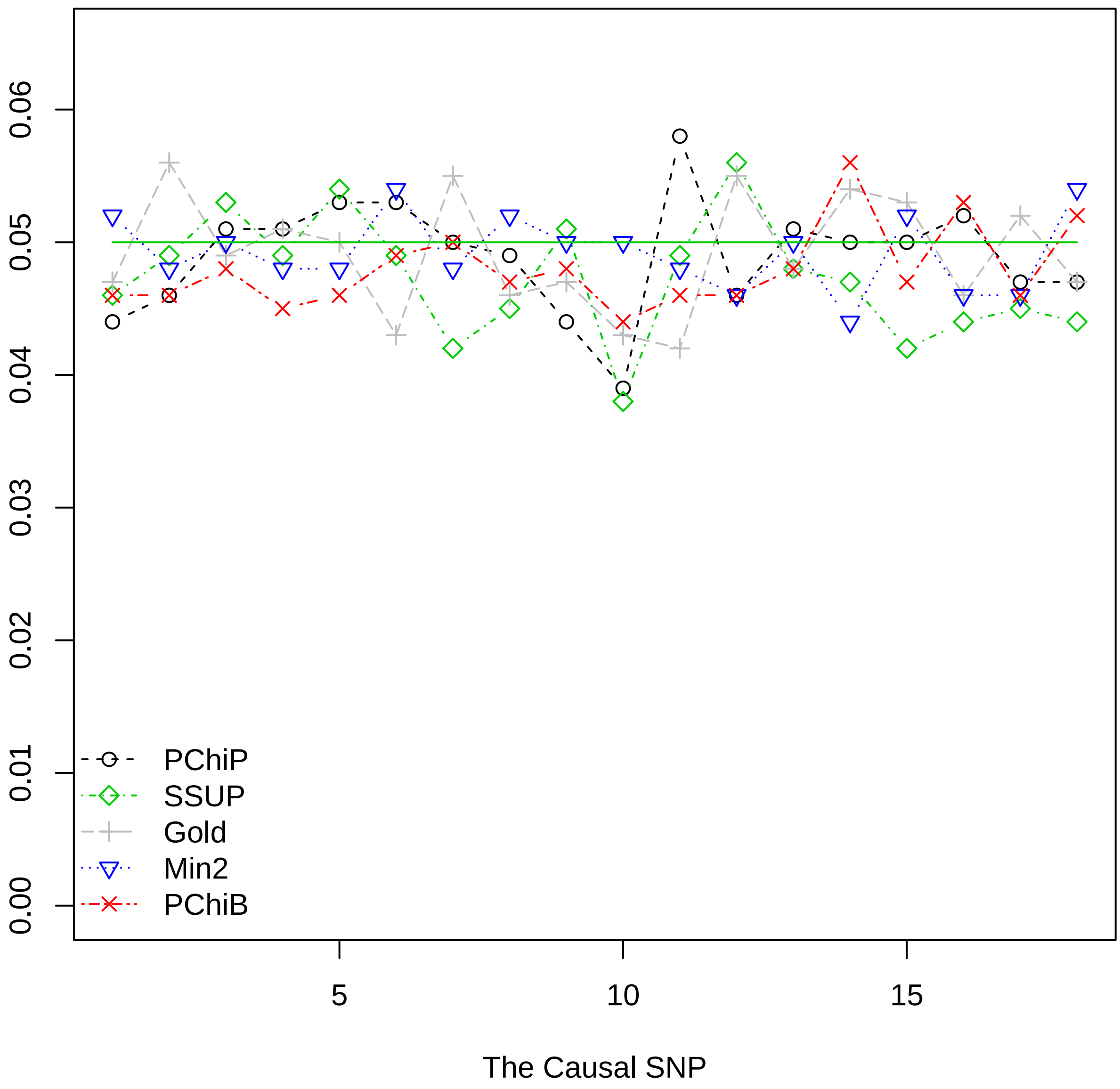

Figure 3a. Empirical null hypothesis rejection rates(based on 17 SNPs excluding the causal SNP) of GOLD, PChiP, SSUP, PChiB and Min2. Each SNP is treated as the causal locus in turn, which has a additive effect with simulated odds ratios 1.0 and Fst=0.5log(2.0) based on 1000 controls, 1000 cases and 500 iterations.

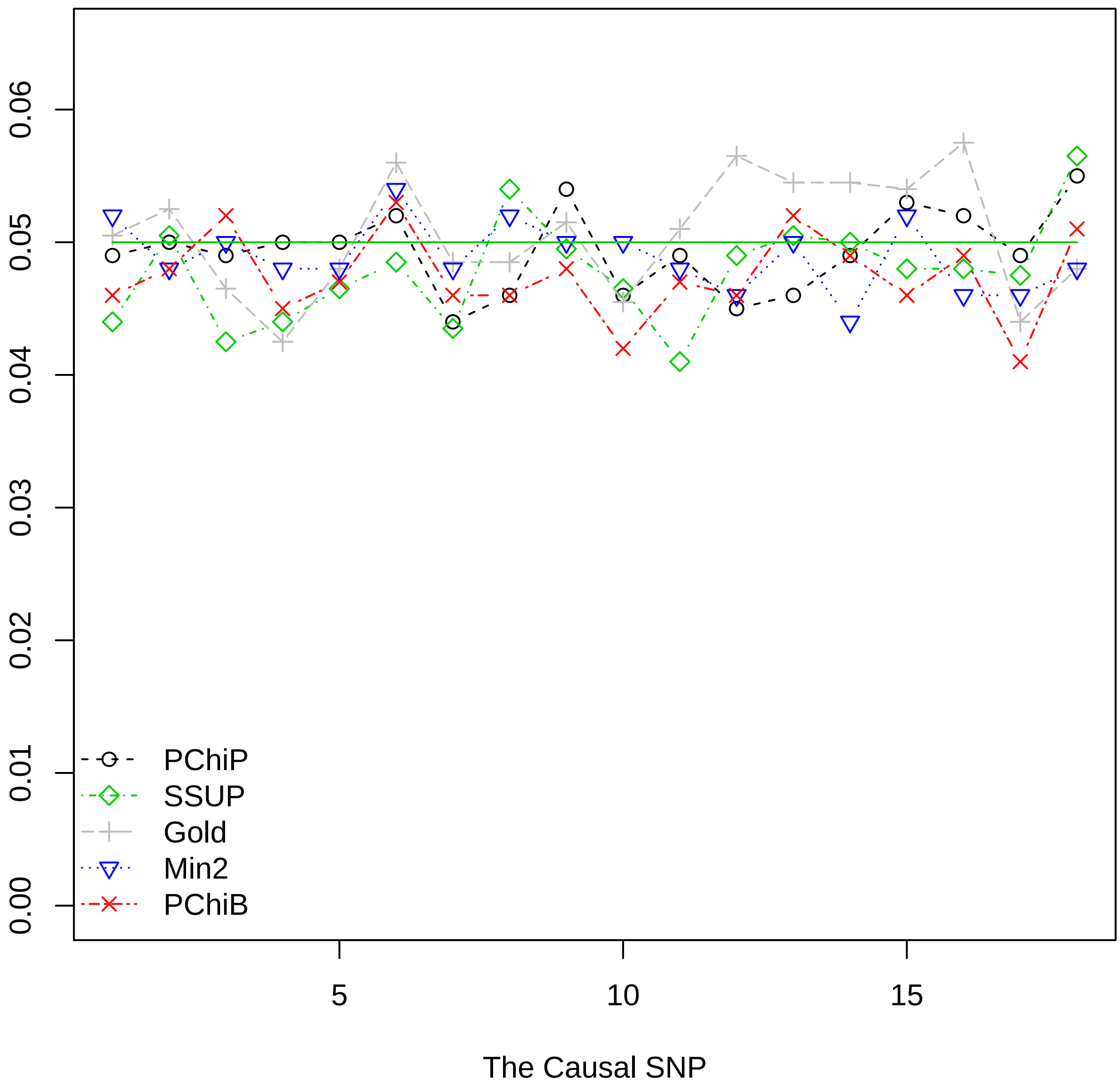

Figure 3b. Empirical null hypothesis rejection rates(based on 5 tag labeled SNPs) of GOLD, PChiP, SSUP, PChiB and Min2. Each SNP is treated as the causal locus in turn, which has a additive effect with simulated odds ratios 1.0 and Fst=0.5log(2.0) based on 1000 controls, 1000 cases and 500 iterations.

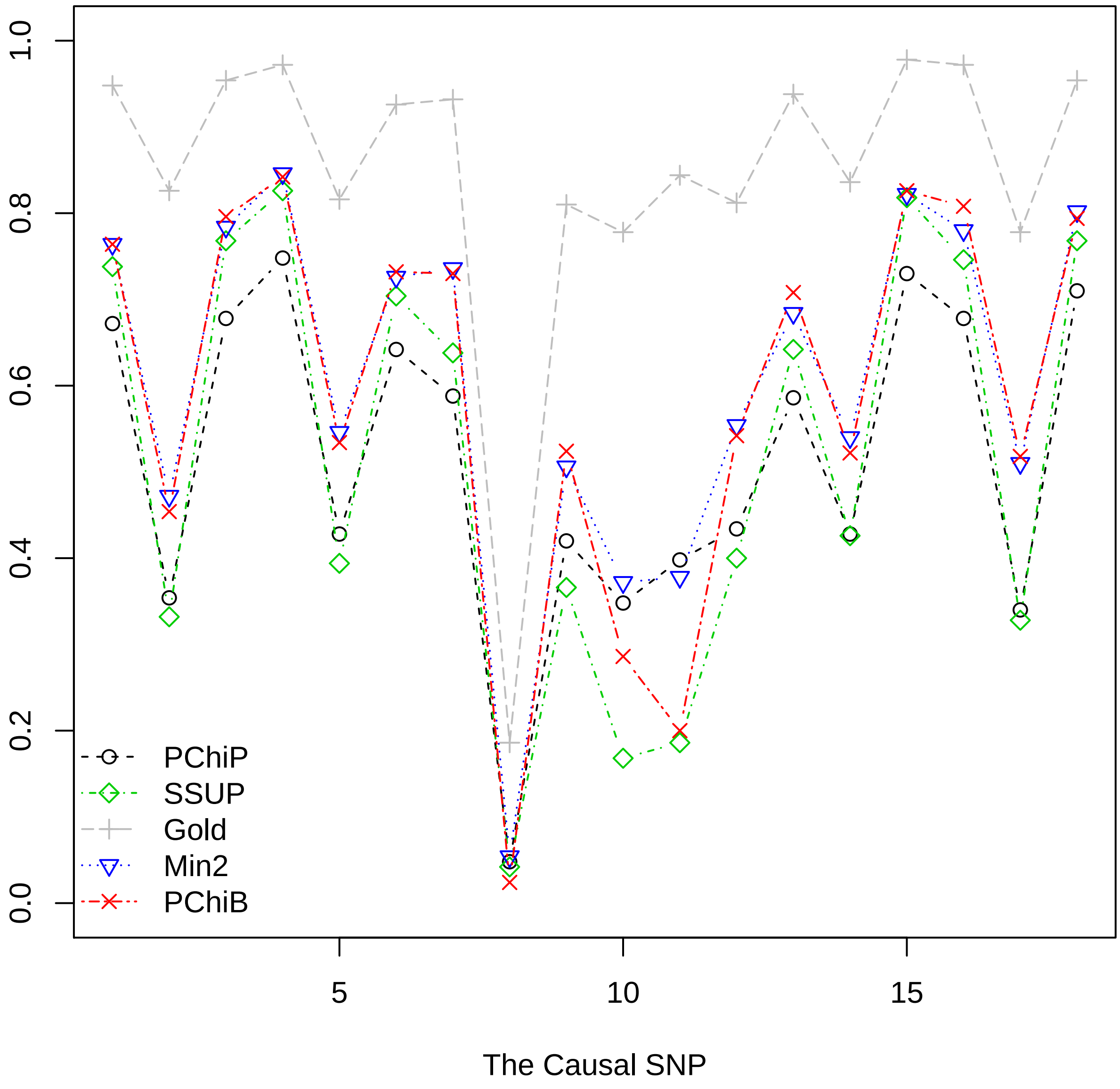

Figure 4a. Empirical powers (based on 17 SNPs excluding the causal SNP) of GOLD, PChiP, SSUP, PChiB and Min2. Each SNP is treated as the causal locus in turn, which has a recessive effect with simulated odds ratios 1.5 and Fst=0 based on 1000 controls, 1000 cases and 500 iterations.

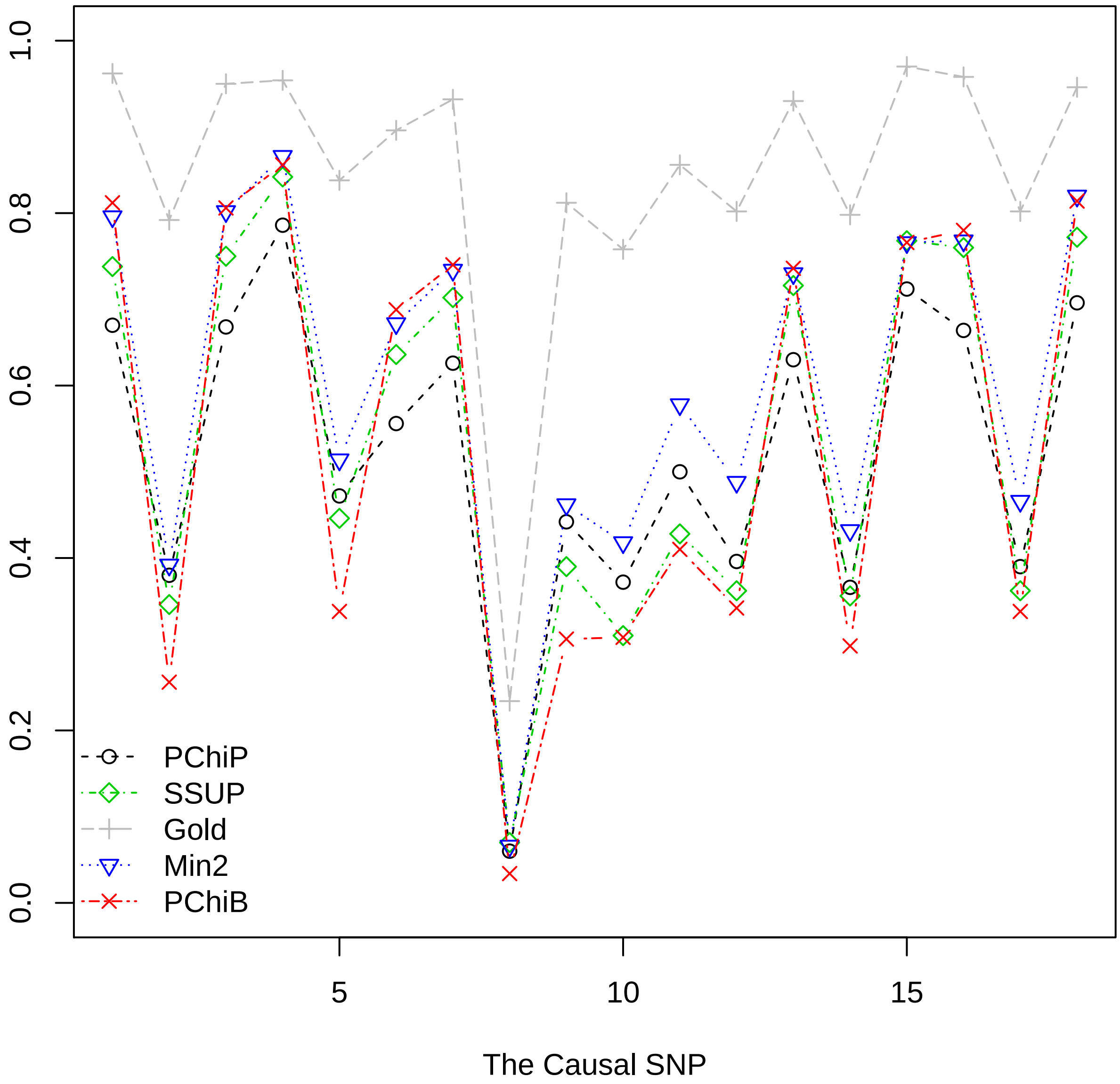

Figure 4b. Empirical powers(based on 5 tag labeled SNPs) of GOLD, PChiP, SSUP, PChiB and Min2. Each SNP is treated as the causal locus in turn, which has a recessive effect with simulated odds ratios 1.5 and Fst=0 based on 1000 controls, 1000 cases and 500 iterations.

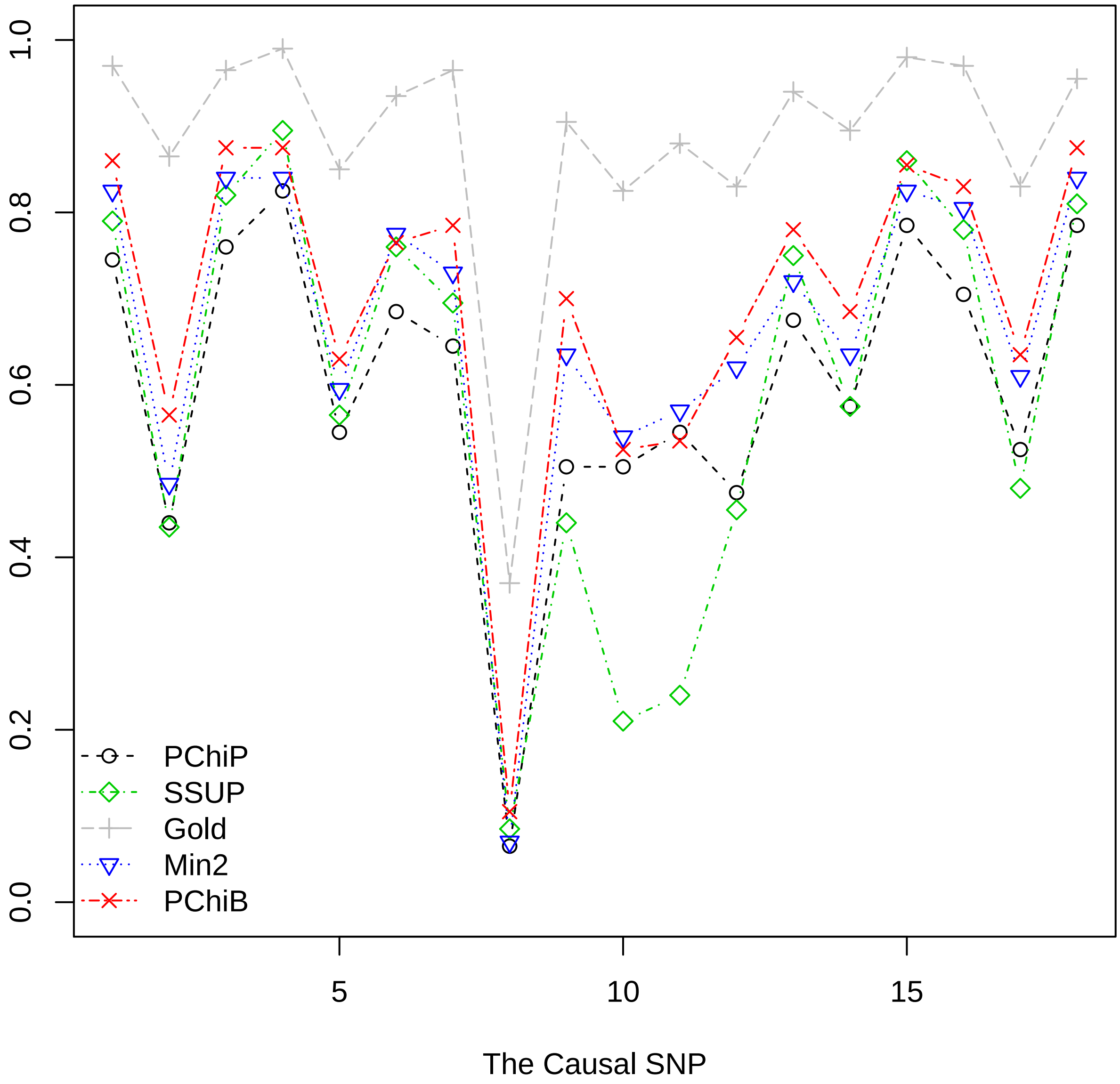

Figure 5a. Empirical powers(based on 17 SNPs excluding the causal SNP) of GOLD, PChiP, SSUP, PChiB and Min2. Each SNP is treated as the causal locus in turn, which has a recessive effect with simulated odds ratios 1.5 and Fst=0.5log(2.0) based on 1000 controls,1000 cases and 500 iterations.

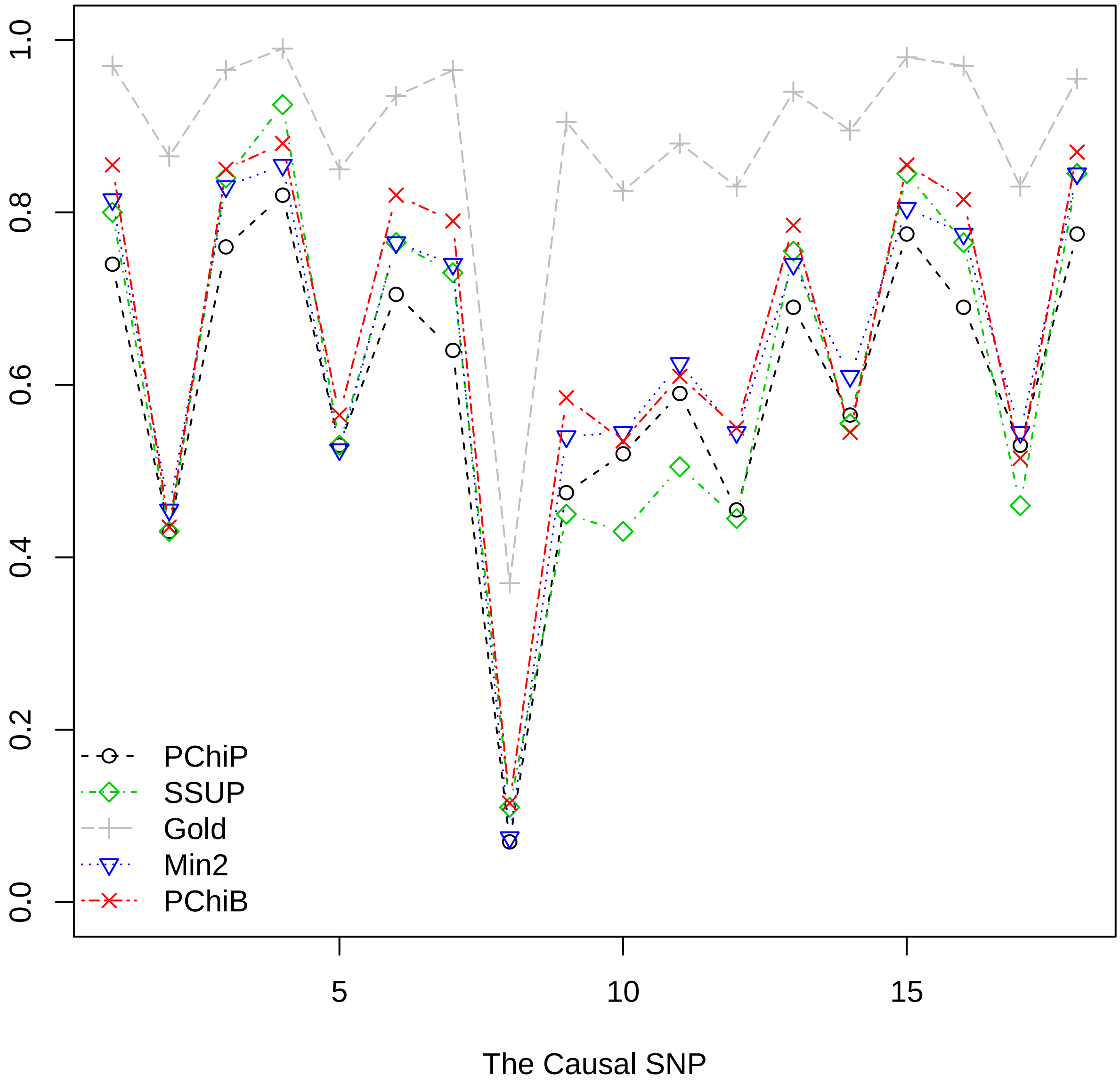

Figure 5b. Empirical powers(based on 5 tag labeled SNPs) of GOLD, PChiP, SSUP, PChiB and Min2. Each SNP is treated as the causal locus in turn, which has a recessive effect with simulated odds ratios 1.5 and Fst=0.5log(2.0) based on 1000 controls,1000 cases and 500 iterations.

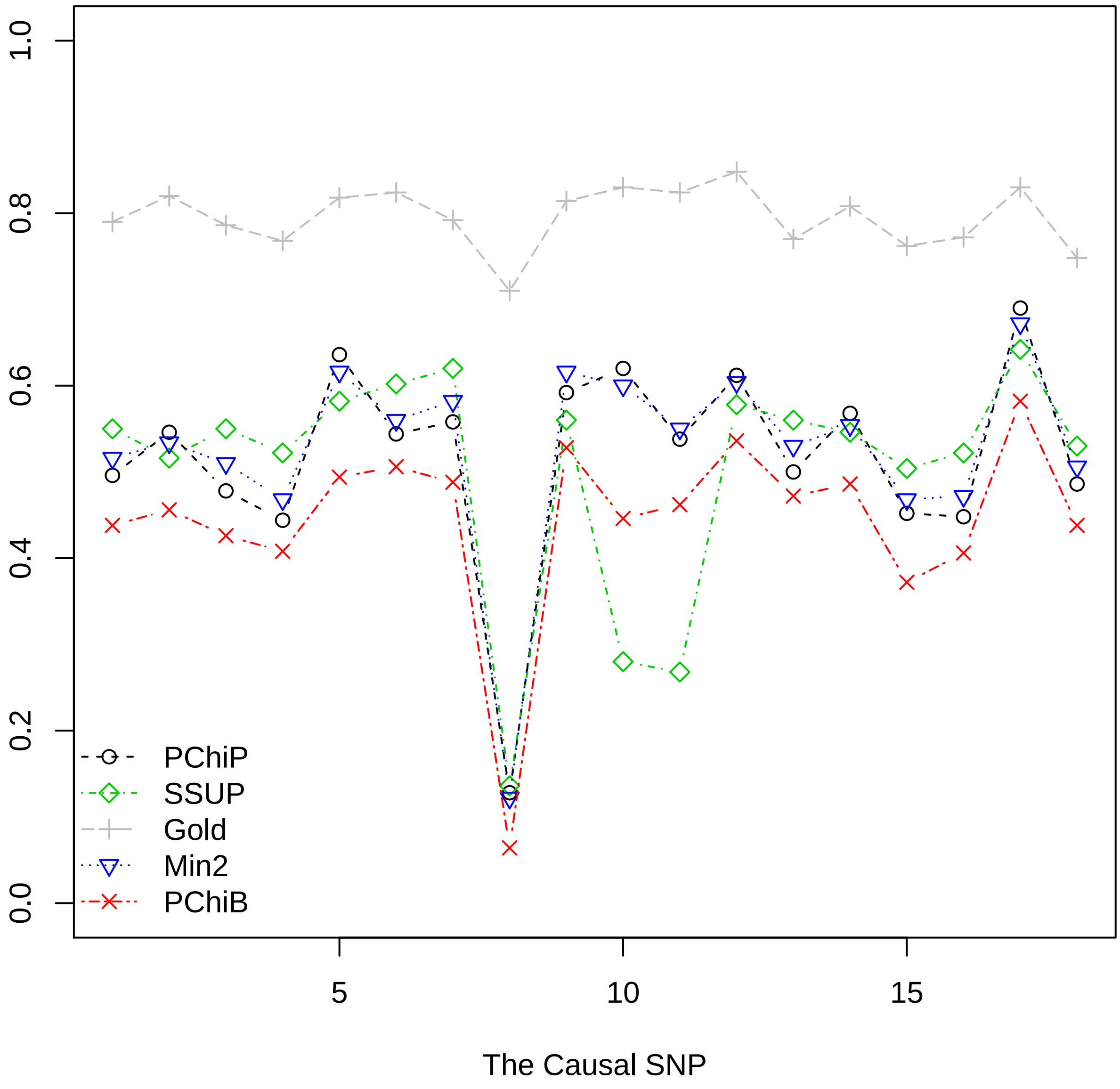

Figure 6a. Empirical powers (based on 17 SNPs excluding the causal SNP) of GOLD, PChiP, SSUP, PChiB and Min2. Each SNP is treated as the causal locus in turn, which has a dominant effect with simulated odds ratios 1.3 and Fst=0 based on 1000 controls, 1000 cases and 500 iterations.

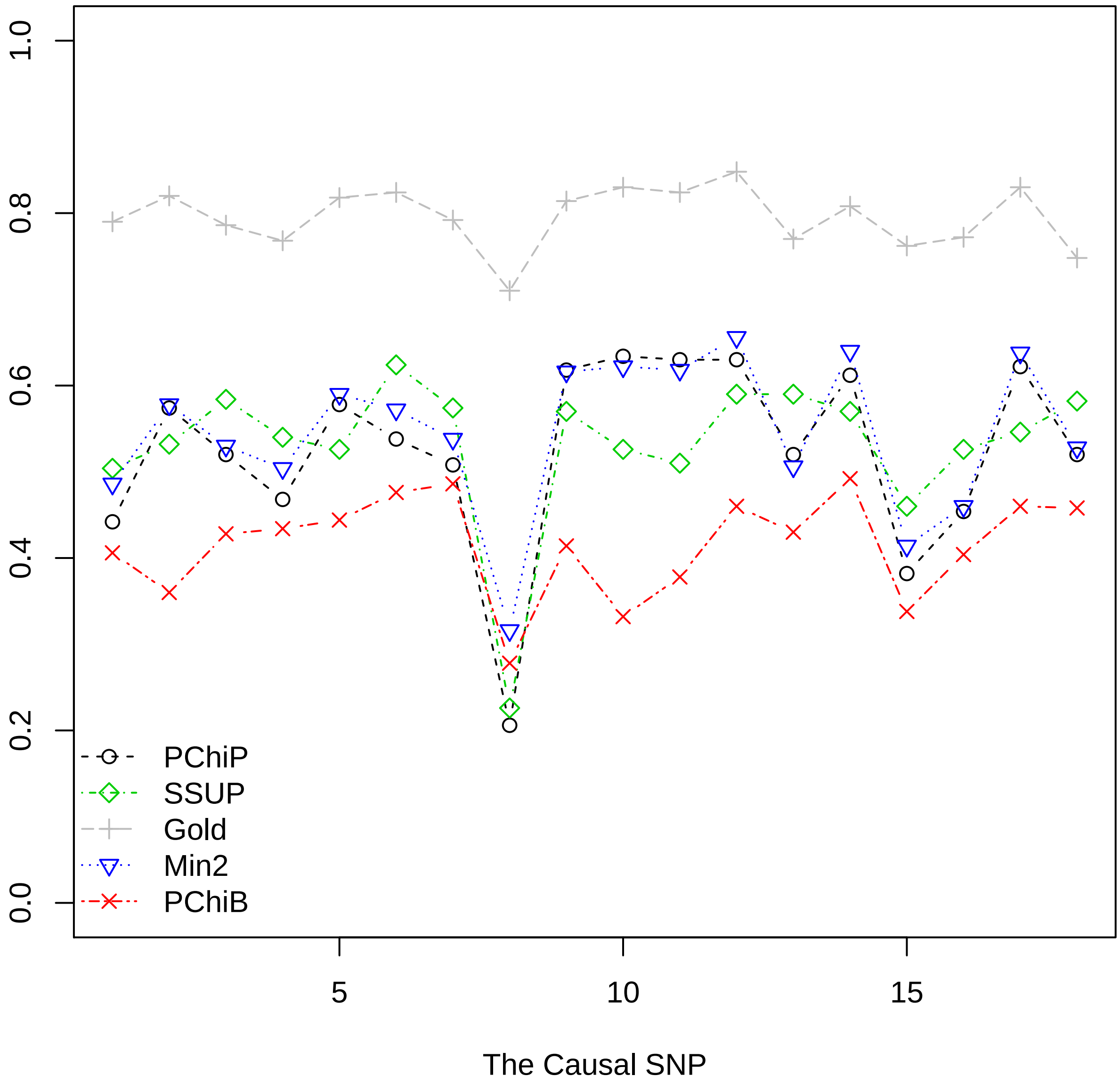

Figure 6b. Empirical powers(based on 5 tag labeled SNPs) of GOLD, PChiP, SSUP, PChiB and Min2. Each SNP is treated as the causal locus in turn, which has a dominant effect with simulated odds ratios 1.3 and Fst=0 based on 1000 controls, 1000 cases and 500 iterations.

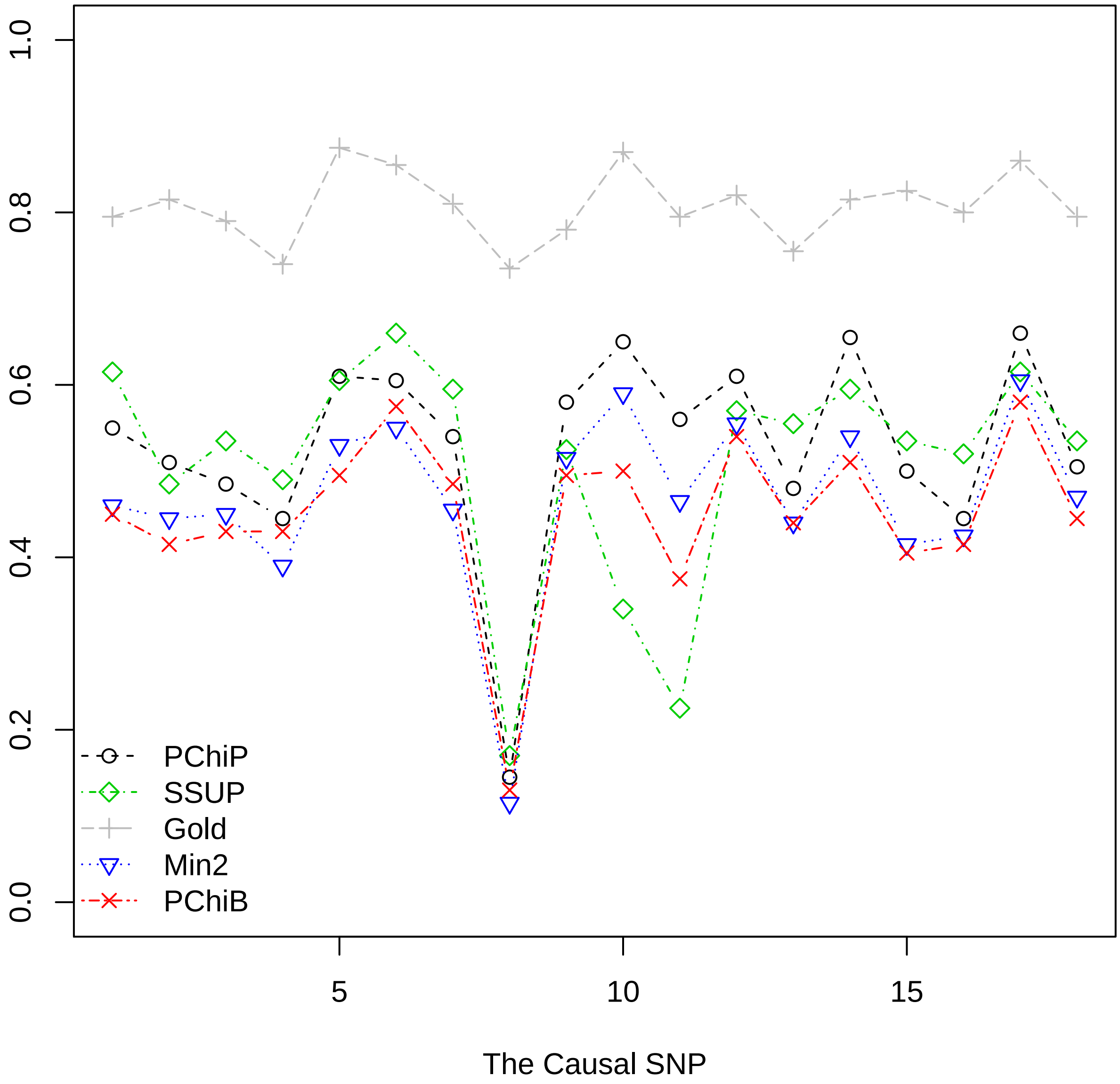

Figure 7a. Empirical powers(based on 17 SNPs excluding the causal SNP) of GOLD, PChiP, SSUP, PChiB and Min2. Each SNP is treated as the causal locus in turn, which has a dominant effect with simulated odds ratios 1.3 and Fst=0.5log(2.0) based on 1000 controls,1000 cases and 500 iterations.

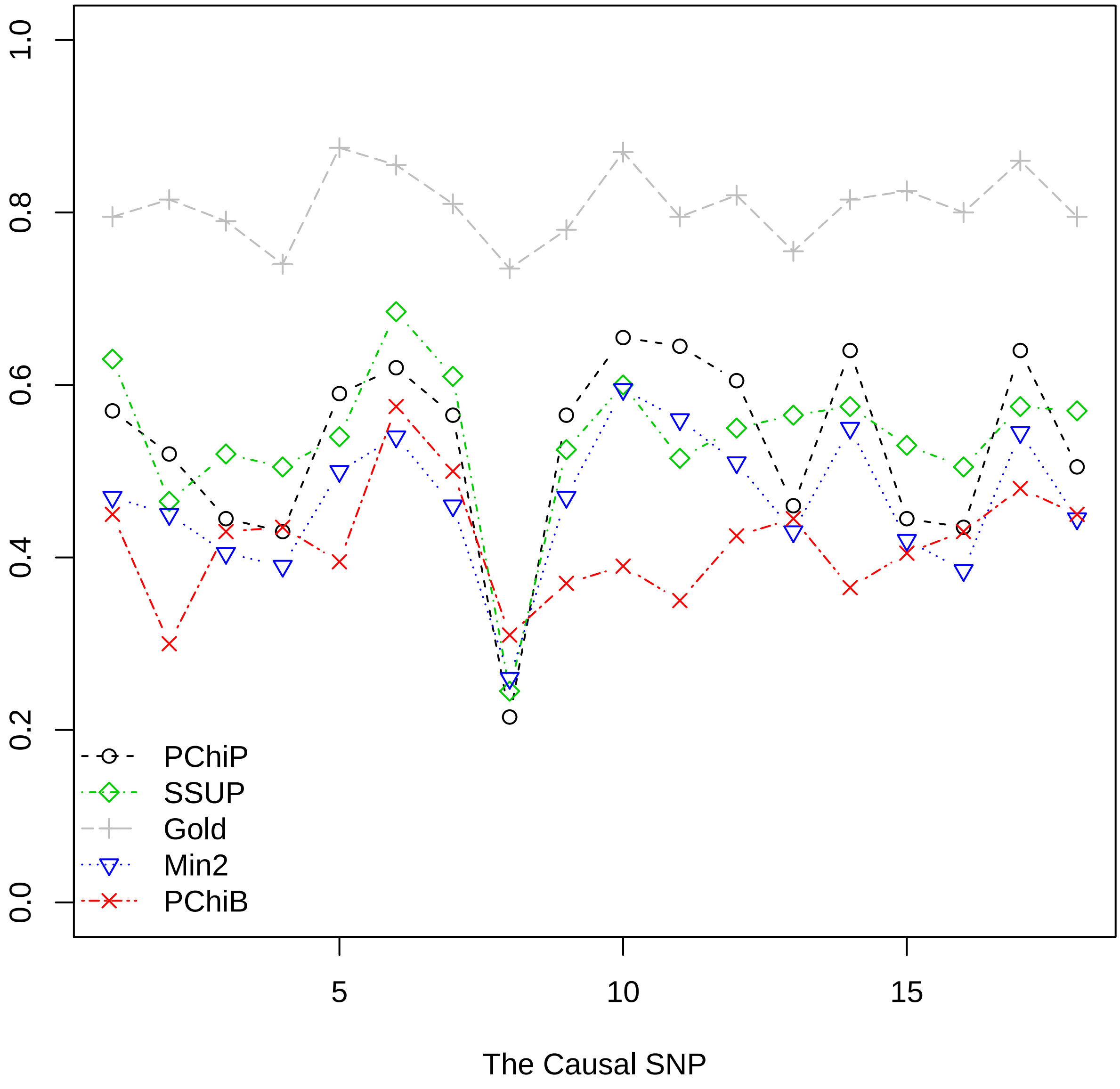

Figure 7b. Empirical powers(based on 5 tag labeled SNPs) of GOLD, PChiP, SSUP, PChiB and Min2. Each SNP is treated as the causal locus in turn, which has a dominant effect with simulated odds ratios 1.3 and Fst=0.5log(2.0) based on 1000 controls,1000 cases and 500 iterations.

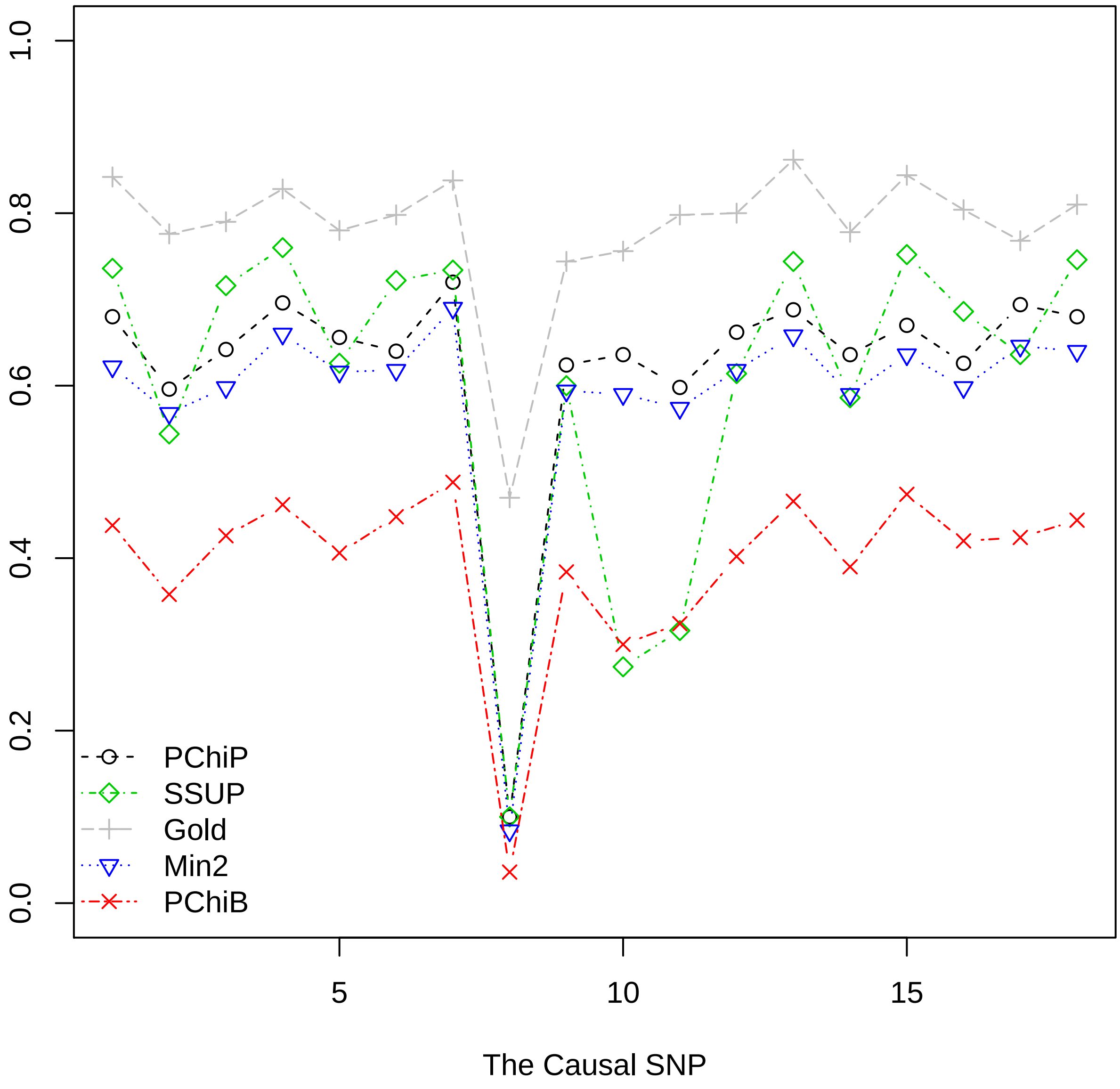

Figure 8a. Empirical powers (based on 17 SNPs excluding the causal SNP) of GOLD, PChiP, SSUP, PChiB and Min2. Each SNP is treated as the causal locus in turn, which has a multiplicative effect with simulated odds ratios 1.2 and Fst=0 based on 1000 controls, 1000 cases and 500 iterations.

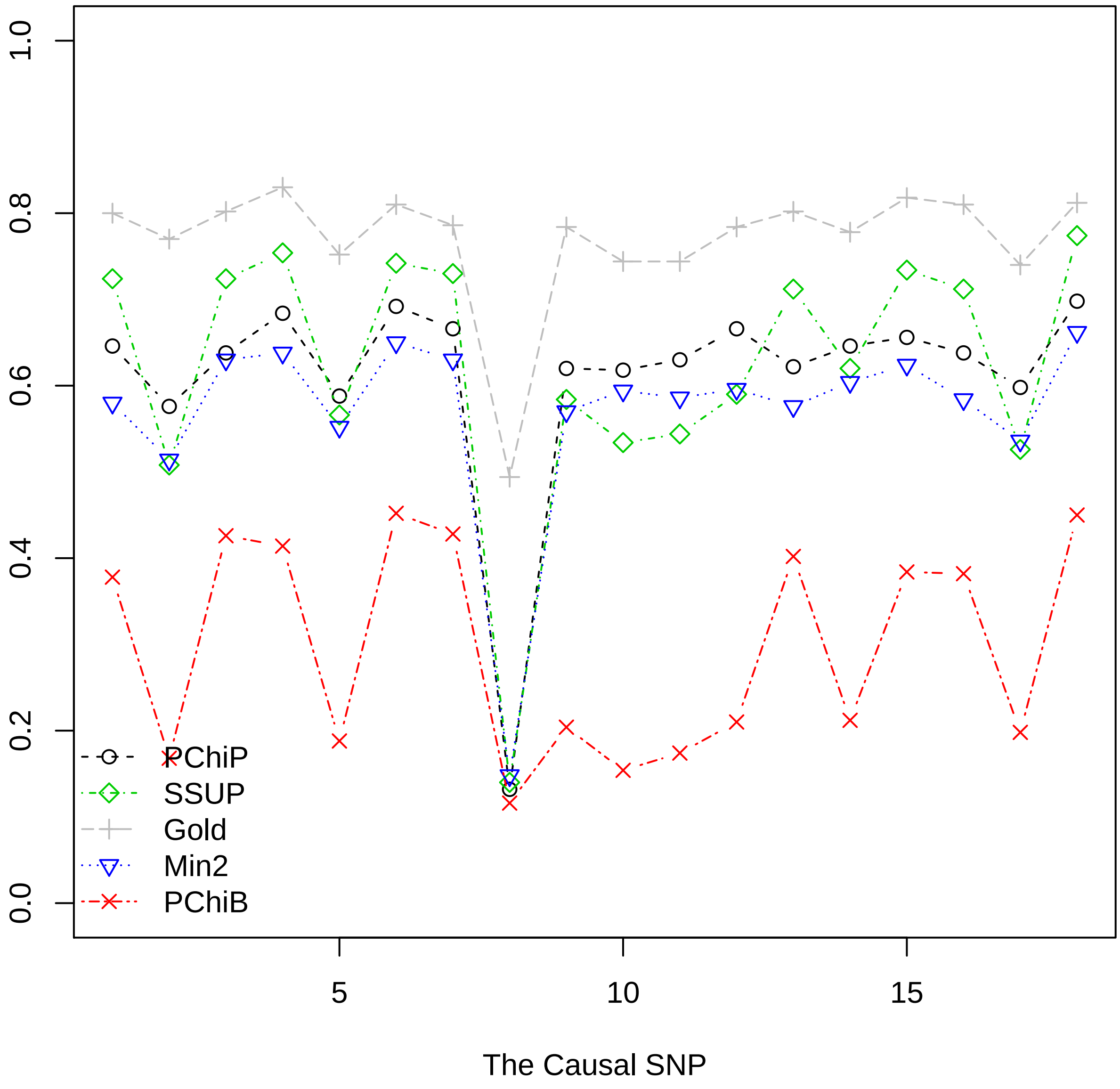

Figure 8b. Empirical powers(based on 5 tag labeled SNPs) of GOLD, PChiP, SSUP, PChiB and Min2. Each SNP is treated as the causal locus in turn, which has a multiplicative effect with simulated odds ratios 1.2 and Fst=0 based on 1000 controls, 1000 cases and 500 iterations.

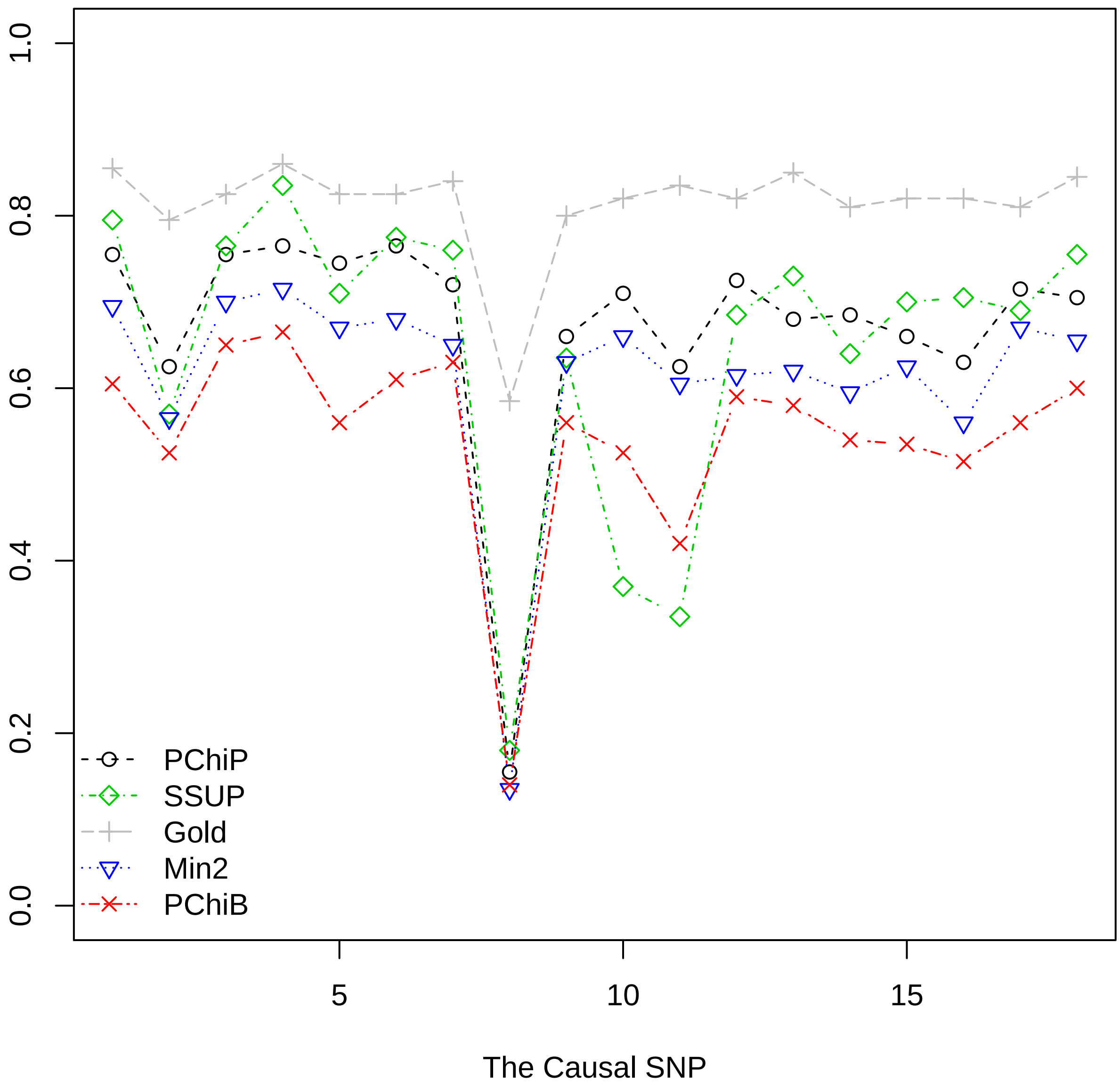

Figure 9a. Empirical powers(based on 17 SNPs excluding the causal SNP) of GOLD, PChiP, SSUP, PChiB and Min2. Each SNP is treated as the causal locus in turn, which has a multiplicative effect with simulated odds ratios 1.2 and Fst=0.5log(2.0) based on 1000 controls,1000 cases and 500 iterations.

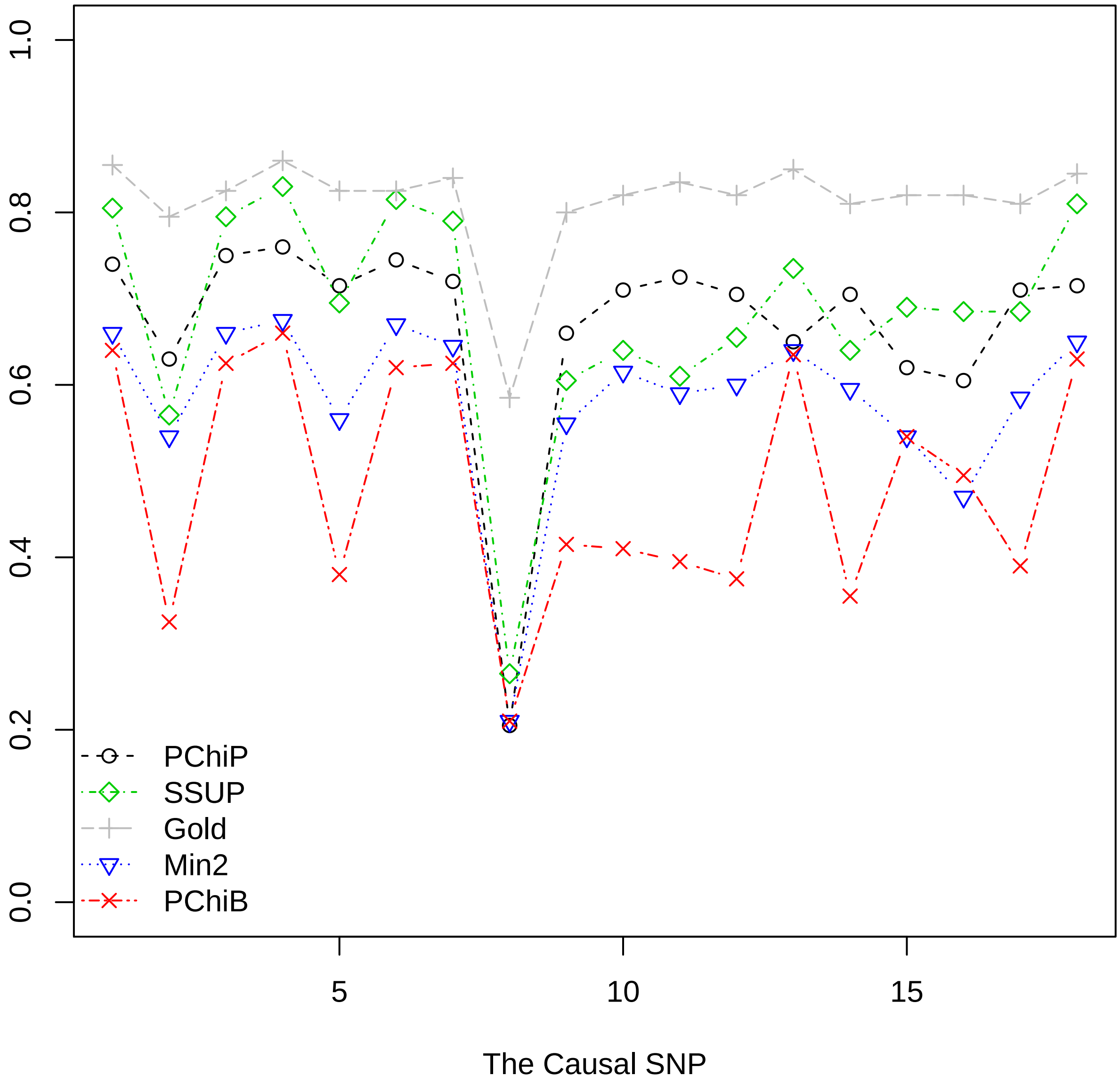

Figure 9b. Empirical powers(based on 5 tag labeled SNPs) of GOLD, PChiP, SSUP, PChiB and Min2. Each SNP is treated as the causal locus in turn, which has a multiplicative effect with simulated odds ratios 1.2 and Fst=0.5log(2.0) based on 1000 controls,1000 cases and 500 iterations.
